# Food for Mood: Relevance of Nutritional Omega-3 Fatty Acids for Depression and Anxiety

**DOI:** 10.3389/fphys.2018.01047

**Published:** 2018-08-06

**Authors:** Thomas Larrieu, Sophie Layé

**Affiliations:** UMR 1286, NutriNeuro: Laboratoire Nutrition et Neurobiologie Intégrée, Institut National de la Recherche Agronomique, Université de Bordeaux, Bordeaux, France

**Keywords:** omega-3 fatty acid, endocannabinoids, HPA axis, nutrient sensing, mood disorders, anxiety, depression, DHA

## Abstract

The central nervous system (CNS) has the highest concentration of lipids in the organism after adipose tissue. Among these lipids, the brain is particularly enriched with polyunsaturated fatty acids (PUFAs) represented by the omega-6 (ω6) and omega-3 (ω3) series. These PUFAs include arachidonic acid (AA) and docosahexaenoic acid (DHA), respectively. PUFAs have received substantial attention as being relevant to many brain diseases, including anxiety and depression. This review addresses an important question in the area of nutritional neuroscience regarding the importance of ω3 PUFAs in the prevention and/or treatment of neuropsychiatric diseases, mainly depression and anxiety. In particular, it focuses on clinical and experimental data linking dietary intake of ω3 PUFAs and depression or anxiety. In particular, we will discuss recent experimental data highlighting how ω3 PUFAs can modulate neurobiological processes involved in the pathophysiology of anxiety and depression. Potential mechanisms involved in the neuroprotective and corrective activity of ω3 PUFAs in the brain are discussed, in particular the sensing activity of free fatty acid receptors and the activity of the PUFAs-derived endocannabinoid system and the hypothalamic–pituitary–adrenal axis.

## Introduction

**FIGURE 1:**
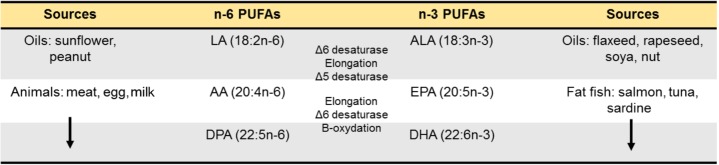
Long-chain PUFAs synthesis. Essential fatty acids precursors of n-6 and n-3 PUFAs are provided by food. Once in the livers, they are metabolized into long-chain PUFAs using a series of desaturations and elongation machinery. The newly synthetized long-chain n-6 PUFAs are AA (20:4n-6) and DPA (22:5n-6) and the long-chain n-3 PUFAs are DHA (22:6n-3) and DPA (22:5n-3).

Since the discovery of omega-3 (ω3) PUFAs in 1929 by George Burr and Mildred Burr (Burr and Burr, 1929; Spector and Kim, 2015), research on ω3 PUFAs became an appealing topic ranging from their role in cardiovascular risk to more recently neuropsychiatric pathologies such as depression and anxiety, cognitive decline or neurodegenerative diseases (Bazinet and Layé, 2014; Joffre et al., 2014; Coulombe et al., 2017). The relevance of lipids in brain function is illustrated by the fact that the CNS has the highest concentration of lipids in the organism after the adipose tissue (50–60% of the dry weight of the brain; Sastry, 1985). Among these lipids, the brain is particularly greedy for PUFAs from the ω6 and ω3 PUFAs families, in particular the LC PUFA (AA, 20:4n-6) and (DHA, 22:6n-3), respectively (Sastry, 1985). In the Human brain, DHA accounts for 10 to 15% of the total fatty acids (saturated, monounsaturated and PUFAs) in both males and females (McNamara et al., 2007, 2008b). This makes PUFAs indispensable to the normal development and function of the CNS (Makrides and Gibson, 2000; Innis, 2007; Bazinet and Layé, 2014). One hypothesis explaining this abundance in brain tissue is that *Homo sapiens* in Paleolithic settled around the lakes and seas where access to foods rich in ω3 PUFAs is easy (Bradbury, 2011). It is generally considered that humans evolved on a diet with a ratio of ω6 to ω3 PUFAs equal approximately to 1. During the industrial era, the rapid expansion of Western countries has been associated with drastic changes in the ω6/ω3 PUFAs content of the diet. This is reflected in large quantities of ω6 PUFA-containing foods and smaller amounts of ω3 PUFA-rich foods leading to Western diet being typically poor in ω3 PUFAs. In addition, the intake of saturated fats from lard and butter has been replaced by plant-based PUFAs based on recommendations from health agencies (Gibson et al., 2011). As a result, the use of oils such as sunflower oil which are mostly high in (LA, the precursor of AA) and low in a-linolenic acid (ALA, the precursor of DHA) leads to a marked increase in LA intake. In mammals, LA and ALA cannot be synthesized *de novo* and need to be provided through the diet (Simopoulos, 1991; Gibson and Makrides, 2001). These essential PUFAs are metabolized into LC PUFAs using the same enzymatic pathway, meaning that LA and ALA are in competition for endogenous conversion to their respective LC forms AA, and DHA (**Figure 1**) but also for their entry into the brain (Bazinet and Layé, 2014). Of importance, ALA bioconversion into DHA through several cycles of elongation (ELOVLs) and desaturation (Δ5 and Δ6 desaturases) is in the range of 0.05% (Burdge et al., 2003) to 4% (Emken et al., 1994) and might not be sufficient to cover brain needs. This led to the recommendation of dietary intake of oily fish rich in the LC ω3 PUFAs DHA and EPA (Tejera et al., 2016). Overall, western diets which are rich in LA (coming from vegetable oils rich in LA) and poor in ALA and DHA (coming from fat fish, sea food or certain algae) have created “a conditional essentiality for ω3 PUFAs” as previously described by Cunnane (2003) and Gibson et al. (2011). Indeed, the amount of LC ω3 PUFAs needed to compensate this lack in western diet is likely to increase, which is not sustainable in the actual context of fish stock decline (Fernandes and Cook, 2013).

The reduced dietary supply of ω3 PUFAs to the brain is associated with many brain diseases, including depression and anxiety disorders (see review from Müller et al., 2015). Epidemiological studies have linked low ω3 PUFAs dietary intake with the prevalence of depression in the general population (Hibbeln, 1998). Clinical studies further revealed that subjects diagnosed with depression or anxiety display significant lower levels of ω3 PUFAs and higher ratio of ω6 to ω3 PUFAs in the blood and in the brain (Green et al., 2006; McNamara and Liu, 2011; Parletta et al., 2016). Supporting clinical observations, preclinical studies conducted in rodents showed that ω3 PUFA deficient diet consumption induces depressive- and anxiety-like symptoms as well as abnormal social behavior in adult offspring (Lafourcade et al., 2011; Larrieu et al., 2012, 2014, 2015; Bondi et al., 2014). Importantly, the use of dietary animal models has been crucial to study the neurobiological mechanisms underlying the alteration of emotional behaviors following decreased bioavailability of ω3 PUFAs in the brain. In this review, we first discuss clinical and pre-clinical evidence of the importance of ω3 PUFAs in anxiety and depressive disorders as well as the rationale for evaluating baseline levels of ω3 PUFAs prior to starting nutritional intervention studies. Then we describe mechanisms linking ω3 PUFAs and emotional behaviors disturbance, especially the sensing activity of FFAR, the eCB system, glucocorticoids as well as neuroinflammatory pathways.

## The Role of ω3 Pufas in Depression and Anxiety Disorders

### Clinical and Epidemiological Evidence Linking ω3 PUFAs, Depression and Anxiety

Several clinical and epidemiological studies highlighted the link between mood disorders and blood and/or cellular membrane PUFAs content (reviewed in Müller et al., 2015). These observations led to the “phospholipids hypothesis” according to which PUFAs are possible aetiological factors in the development of depressive disorders (Hibbeln and Salem, 1995). Indeed, subjects diagnosed for anxiety and depressive disorders show lower ω3 PUFAs and higher ratio of ω6 to ω3 PUFAs in their blood and brains compared to healthy subjects matching for age and sex (Adams et al., 1996; Maes et al., 1996; Edwards et al., 1998a,b; Tiemeier et al., 2003; Frasure-Smith et al., 2004; Green et al., 2006; McNamara et al., 2007; McNamara and Liu, 2011; Parletta et al., 2016). EPA (20:5 n-3) concentration (Adams et al., 1996; Green et al., 2006; Liu et al., 2013) as well as DHA concentration (Edwards et al., 1998a; Frasure-Smith et al., 2004; Green et al., 2006; McNamara and Liu, 2011; Liu et al., 2013; Otoki et al., 2017) are decreased in the membrane of erythrocytes and in the plasma of patients suffering from unipolar depression, seasonal winter affective disorder or social anxiety disorders (Adams et al., 1996; Green et al., 2006). A recent study showed that the AA:EPA ratio in the blood is positively correlated with illness duration in patients diagnosed with major depression (Scola et al., 2018). A meta-analysis work from Lin et al. (2010) supports these observations by showing significant low levels of EPA, DHA, and total ω3 PUFAs among 3,318 depressed patients. In some of these studies, the severity of the depressive and anxious symptoms is negatively correlated with the concentration of the total ω3 PUFA levels in the blood (Adams et al., 1996; Maes et al., 1996; Edwards et al., 1998a; Green et al., 2006; Liu et al., 2013). Post-mortem studies report reduced levels of DHA in the PFC of patients diagnosed with major depression, bipolar disorders or committing suicide (McNamara et al., 2007, 2008a, 2013). In pregnant women, for whom the risk of ω3 PUFAs deficiency is relatively high as they provide DHA to the fetus, nearly 10% experience post-partum depression (Markhus et al., 2013). Yet, no clear consensus exist as to whether low ω3 PUFAs and high ω6 PUFA levels in the blood are linked to the development of post-partum depression (Hibbeln, 2002; Parker et al., 2015). These observations are not limited to depression as decreased ω3 PUFAs (DHA) levels in erythrocytes and PFC have also been found in patients suffering from PTSD (de Vries et al., 2016). As decreased ω3 PUFAs status is associated with several forms of depression and stress disorders, the understanding of the origin (dietary or genetic) of the decreased bioavailability of DHA or EPA is of high interest.

The rationale for identifying baseline nutritional ω3 PUFAs status comes from one hypothesis advanced by researchers that weak food supply in ω3 PUFAs might be a risk factor of the development of depression. As fish is the main source of LC ω3 PUFAs, several epidemiological studies investigating putative associations between major depressive disorder and fish consumption were conducted in various countries (Finland, New Zealand, France, Northern Ireland, Norway or Netherlands) (Tanskanen et al., 2001; Silvers and Scott, 2002; Timonen et al., 2004; Barberger-Gateau et al., 2005; Kamphuis et al., 2006; Appleton et al., 2007; Raeder et al., 2007; Colangelo et al., 2009). Subjects having low fish consumption (lower than once per week, including seafood) present high scores of depression (Timonen et al., 2004; Barberger-Gateau et al., 2005). A transnational ecological study conducted on a large cohort including individuals from different countries highlighted a strong negative correlation between fish consumption and the prevalence of depression (Hibbeln, 1998). Indeed, this epidemiological study of great width (170,000 individuals) revealed that individuals from Asian countries like Japan, Korea, and Taiwan, who are the biggest fish consumers, suffer relatively little from major depression. This observation can appear counterintuitive as Japan experiences a high rate of suicide while the depression rate is thought very low (WHO, 2015). Such a high rate of suicide has been associated to cultural factors (idealization of suicide, acceptability, etc.) including aging society (Saito et al., 2013), divorce and unemployment (Yamauchi et al., 2013). On the contrary, Western countries like New Zealand, Canada, United States, Germany, or France are part of the countries that consume less fish with high prevalence to develop depression. These data suggest that fish consumption is conversely correlated with the development of depression.

To PUFA dietary intake consideration in decreasing ω3 PUFAs bioavailability in depression, one must add the genetic variation of the FADS, an enzyme which converts PUFA precursors into LC-PUFAs (EPA, DHA, and AA) (Koletzko et al., 2011; Mathias et al., 2014; Park et al., 2015). Indeed, inter-individual variability in red blood cells DHA and AA levels is explained by FADS polygenes (71 and 53%, respectively) (Lemaitre et al., 2008). FADS genotypes influence DHA amounts in red blood cells of pregnant women independently of dietary effects (Koletzko et al., 2011). Children carrying FADS minor allele have lower DHA levels in erythrocyte, with no behavioral outcomes (Jensen et al., 2014). However, the link between FADS haplotype and the risk of developing neuropsychiatric disorders (schizophrenia or depression) is weak (Fallin et al., 2004; Schizophrenia Psychiatric Genome-Wide Association Study (GWAS) Consortium, 2011). A study conducted in patients with major depression found no association between FADS single nucleotide polymorphisms and major depression (Sublette et al., 2016). Recently, a study suggested that genetic variation in the FADS gene influences the ω6/ω3 PUFAs ratio which appears to be associated with major depression (Cribb et al., 2017). To our knowledge, no study has so far linked brain DHA level to FADS genotype. Overall, in addition to dietary composition of PUFAs, FADS genetic variation should be considered in the LC-PUFAs status and the pathophysiology of depression and stress.

Taken together, these clinical observations raise a crucial question: Is there a causal link between the contents of ω3 PUFAs in the blood/brain and depressive/anxiety disorders? If so, are the low levels of ω3 PUFAs the cause or the consequence of these affective disorders? These relations of causalities were approached by nutritional interventions in Humans which are the object of the following section, first in depression, then in PTSD and stress disorders.

### Dietary ω3 PUFAs Supplementation, Depression, and PTSD in Humans

The results from LC ω3 PUFA nutritional interventions carried out among patients with depressive disorders are heterogeneous as recently reviewed elsewhere (Bozzatello et al., 2016; Saunders et al., 2016). Some studies conducted on patients suffering from major depression without an antidepressant treatment do not show significant effects of ω3 PUFAs supplementation (Marangell et al., 2003; Freeman et al., 2008; Rees et al., 2008; Mischoulon et al., 2015), whereas others reveal a beneficial effect (Su et al., 2003; Nemets et al., 2006; Jazayeri et al., 2008). A 16-week dietary supplementation with EPA + DHA did not prevent maternal depressive symptoms (Vaz et al., 2017). These discrepancies are reflected by meta-analysis. Indeed, some found that EPA and DHA can reduce depressive symptoms (Kraguljac et al., 2009; Appleton et al., 2010; Sublette et al., 2011; Grosso et al., 2014) while other found no effect (Appleton et al., 2015). Mixed results of clinical trials could be attributed not only to the heterogeneity in clinical trials and design, but also to the quantity and quality of the PUFA used, including the EPA:DHA ratio, trial duration, the type of placebo (PUFA or other fatty acids) and to the concomitant use of medication and baseline symptom severity. Recently, EPA, rather than DHA, has been suggested to mediate the beneficial effect of ω3 PUFAs supplementation in patients diagnosed with major depression DHA (Martins, 2009; Martins et al., 2012; Grosso et al., 2014; Hallahan et al., 2016). Indeed, several studies using ethyl-EPA (from 1 to 2 g/day) reported a beneficial effect in patients with major depression and resistant to anti-depressant (Peet and Horrobin, 2002) or recurrent unipolar depression (Nemets et al., 2002). In addition, EPA-rich formulation with no DHA is more effective than DHA-rich supplements in major depression (Sublette et al., 2011). A meta-analysis aiming at investigating the beneficial role of EPA or DHA supplementation in major depressive disorder found that EPA is more effective than DHA (Grosso et al., 2014). The beneficial effect of EPA has been recently corroborated by a new meta-analysis (Mocking et al., 2016). EPA efficiency could be linked to its conversion into DHA by elongase leading to increased DHA brain bioavailability and decreased LC ω6 PUFAs production (Ganança et al., 2017). Indeed, as the conversion of EPA into DHA compete with the production of n-6 DPA from AA by using the same enzymatic pathway (i.e., FADS and elongases), the supplementation of EPA can simultaneously lead to an increase in DHA and a decrease in n-6 DPA levels that can subsequently improve mood. However, further studies with larger and more homogeneous samples are required to confirm these effects.

In addition, it has been suggested that depressive patients who display low levels of ω3 PUFAs may rather benefit of the LC ω3 PUFAs supplementation (Carney et al., 2016; Messamore and McNamara, 2016). Despite recent advances in understanding the pathophysiology of major depression, approximately 30% of patients remain refractory to multistep antidepressant treatments (Rush et al., 2006a,b). One explanation of this finding could come from individual differences in baseline levels of ω3 PUFAs. To support this idea, a study recently showed that high baseline levels of EPA and DHA in red blood cells of depressive patients predict favorable depression outcomes in patients receiving ω3 PUFAs supplements (Carney et al., 2016). In addition, among patients treated but resistant to antidepressants such as the (SSRI; e.g., Fluoxetine, Paroxetine), the severity of the symptoms of depression decreased in the group supplemented in ω3 PUFAs (Nemets et al., 2002; Peet and Horrobin, 2002; Su et al., 2003; Jazayeri et al., 2008; Gertsik et al., 2012; Zimmer et al., 2013; McNamara et al., 2014; Mocking et al., 2016). This strategy can be highly relevant as resistance to treatment is observed in a large proportion of patients (40%) (Brunoni et al., 2009; Shelton et al., 2010) and it suggests that dietary ω3 PUFA intake may improve antidepressant response.

ω3 PUFAs dietary supplementation has also been used in stress disorders. Stress is a well-known major risk factor for the development of depression or PTSD. One study aimed at investigating the effect of ω3 PUFAs supplementation in chronically work-stressed individuals and found no significant treatment effect for EPA after 12 weeks of supplementation on the Perceived Stress Scale scores (Bradbury et al., 2017). On the contrary, a placebo-controlled trial of ω3 PUFAs supplements in patients suffering from PTSD revealed that EPA but not DHA (Matsuoka et al., 2015) levels were inversely correlated with PTSD severity suggesting the potential efficacy of EPA rather than DHA for minimizing PTSD symptoms (Matsuoka et al., 2016). Regarding ω3 PUFAs supplementation in the prevention of anxiety, a study conducted by Yehuda et al. (2005) has investigated whether administration of a cocktail of ω3 PUFAs (90 mg of ALA/day) and ω6 PUFAs (360 mg of LA/day) over 3 weeks in students could improve anxiety induced by the university examinations. These authors highlighted an improvement of several symptoms (appetite, mood, concentration, and fatigue) compared to the placebo group. These improvements are associated with a decreased level of salivary cortisol (Yehuda et al., 2005). Despite the low dose of ALA used in this study (90 mg/day) as compared to nutritional intake in the general population (1–2 g/day), this 10% increase was sufficient to improve symptoms. In addition, ALA conversion in EPA is determined by the amount of ALA in the diet (i.e., higher in the plasma phospholipid pool when ALA is low) (Goyens et al., 2006). Thus, the effectiveness of ALA in Yehuda et al. (2005) study could be linked to EPA. Moreover, students having received a supplementation with DHA and EPA during 12 weeks present a reduction of 20% of anxiety symptoms compared to the students treated with a placebo (Kiecolt-Glaser et al., 2011). In students treated with ω3 PUFAs supplementation, an increase in plasma concentration of DHA and EPA were observed as of the third week of treatment. Lastly, the increase in DHA and EPA was negatively correlated with a reduction in anxiety symptoms.

In conclusion, these observations further support the role of PUFAs metabolism as an important mechanism in depression and anxiety disorders treatment. This brings new insight to personalized PUFAs formulation as a novel adjunctive treatment for patients with mood and anxiety disorders.

### Pre-clinical Studies Linking ω3 PUFAs and Emotional Behavior

To better understand whether the modifications of nutritional ω6/ ω3 PUFA ratio could affect brain function and behavior, studies have been carried out in animals (rodents, monkeys, and pigs) subjected to diets in which PUFAs contents are controlled during one or several generations (de la Presa Owens and Innis, 1999; Clouard et al., 2015). Numerous studies have shown that in animal models of nutritional ω3 PUFA deprivation, brain DHA levels were decreased while AA levels were increased in several brain areas leading to an imbalance between ω6 and ω3 PUFAs in the ω3 PUFAs deficient mouse brain (Delion et al., 1994; Francès et al., 1995; Favrelière et al., 1998; Carrié et al., 2000b; McNamara and Carlson, 2006; Lafourcade et al., 2011; Larrieu et al., 2012). Nutritional ω3 PUFAs deficiency-induced reduction of brain DHA levels has been associated with the development of depression-like behavior (Carrié et al., 2000a; Takeuchi et al., 2003; DeMar et al., 2006; Fedorova and Salem, 2006; Lafourcade et al., 2011; Larrieu et al., 2012, 2014, 2015; Bondi et al., 2014; Morgese et al., 2016; Manduca et al., 2017). By submitting mice to one generation dietary ω3 PUFAs deficiency, we found that ω3 PUFAs deficient diet alone disturbed social behavior as well as increased anxiety- and depression-related behavior in an open-field and FSTs, respectively (Lafourcade et al., 2011; Larrieu et al., 2012, 2014, 2015). Some studies conducted in rats indicate that the time of immobility in the FST was increased by ω3 PUFAs deficiency (DeMar et al., 2006; Morgese et al., 2016) and reduced by ω3 PUFAs supplementation with fish oil (Naliwaiko et al., 2004; Carlezon et al., 2005; Huang et al., 2008). In addition, the level of DHA in rat whole brains is negatively correlated with the time spent immobile during the FST, a behavioral test used for evaluating the efficacy of compounds rendering or preventing depressive-like states. Interestingly, similar behavioral impairments (e.g., anxiety-like behavior and social interaction) occur in mice after exposure to CSDS, a well-characterized preclinical model of anxiety and depression (Golden et al., 2011; Bosch-Bouju et al., 2016; Larrieu et al., 2017). This model presents strong face validity, as social defeat (e.g., bullying) is a major risk factor to developing depression in humans. One cardinal feature of CSDS is that mice experiencing this chronic stress develop a long-lasting (more than 1 month) aversion to social interaction as well as anhedonia, which can be normalized after chronic (28 days post-CSDS), but not acute administration of antidepressant (Berton et al., 2006; Krishnan et al., 2007) as observed in humans. By comparing the effects of dietary ω3 PUFAs deficiency to those of CSDS on emotional behavior, We found that mice fed with a diet deficient in ω3 PUFAs exhibited behavioral changes and neuronal atrophy profile that resemble those of mice exposed to CSDS (Larrieu et al., 2014). Interestingly, behavioral alterations can be reversed after chronic ω3 PUFAs supplementation. As such, increased anxiety- and depressive-like behavior after chronic stress is normalized after ω3 PUFAs supplementation (Ferraz et al., 2011; Larrieu et al., 2014).

## Relevant Mechanisms for Nutritional ω3 Pufa-Induced Mood-Related Behavioral Deficits

Numerous epidemiological, clinical, and preclinical studies demonstrated the key role of nutritional ω3 PUFAs in depression and anxiety disorders. In recent years, emphasis was made on identifying molecular and cellular mechanisms by which ω3 PUFAs modulate brain function. ω3 PUFAs and their metabolites are well known to play an important role as signaling molecules that regulate inflammation (Serhan, 2014) and neuroinflammation (recently reviewed in Layé et al., 2018). They also contribute to signal transduction between neurons or neurons and glial cells. Here, we will focus on DHA, the most aggregated fatty acid in the brain while EPA is rapidly b-oxidized and poorly accumulated (Chen and Bazinet, 2015). As DHA is poorly synthesized *de novo*, its brain levels depend on both the dietary supply and blood level bioavailability (Bazinet and Layé, 2014; Lacombe et al., 2018). Once free DHA has entered the brain, it is esterified at membrane phospholipids (both in neurons and glial cells). However, upon neuronal stimulation, injury or stress, DHA is released from phospholipids and can either activate specific receptors or be metabolized into specific derivatives, such as eCBs or oxylipins which regulate specific pathways important to neurotransmission or neuroinflammation (Bazinet and Layé, 2014; Bosch-bouju and Layé, 2016; Layé et al., 2018). In the following section, we first describe the receptors which have been reported to mediate DHA effect in the brain. Then, we focus on the regulation of the eCB system and the HPA axis as recent data show that they could mediate the neuroprotective effect of ω3 PUFAs as both are thought to be involved in depression.

### Direct Effect of DHA on Specific Receptors

While free fatty receptors have been widely described to mediate some of the effects of DHA at the periphery, few reports highlight a direct effect of DHA through signaling activity in the brain. In 2000, DHA has been shown to be a ligand of the RXR, the receptor of retinoic acid (a vitamin A metabolite), which heterodimerizes with other nuclear receptors such as retinoic acid receptor, vitamin D receptor, thyroid hormone receptor or PPAR (Lengqvist et al., 2004). DHA effect on neuritogenesis does not involve RXR, as its effect *in vitro* does not activate RXR (Calderon and Kim, 2007). However, DHA potentiates retinoic acid effect and improves cognitive symptoms in a rodent model of Alzheimer disease (Casali et al., 2015) and aged rodents (Létondor et al., 2016). Interestingly, the loss of RXR signaling leads to altered emotional and cognitive behavior in mice (Krzyzosiak et al., 2010; Wietrzych-Schindler et al., 2011). Importantly, DHA antidepressant effect is absent in RXR knock-out mice (Wietrzych-Schindler et al., 2011), further highlighting the role of this receptor and its ligand (possibly DHA and retinoic acid) in emotional behavior. FFAR, members of the “rhodopsin-like” GPCR family, namely GPR40 (FFAR1) and GPR120 (FFAR4), have been recently highlighted as potentially mediating LC FFAs signal from pancreatic beta-cells as well as the intestines (Itoh et al., 2003; Hirasawa et al., 2005). These lipid receptors were also reported to be present in the brain (Ma et al., 2007; Dragano et al., 2017). Memory-induced progenitor cell proliferation and DHA-induced neurogenesis in the hypothalamus are mediated by GPR40 (Ma et al., 2008; Yamashima, 2008; Nascimento et al., 2016). In addition, DHA-induced GPR40 signaling pathway activates β-endorphin release in the hypothalamus of rodents (Nakamoto et al., 2015). Importantly, the chronic activation of GPR40 signaling in the brain reduces depressive-like behavior (Nishinaka et al., 2014). In addition, anxiety-like behavior and sucrose preference, a behavioral sign of anhedonia, are reduced in GPR40 knock-out mice further highlighting the role of GPR40 signaling in the pathophysiology of mood disorders (Aizawa et al., 2016). GPR120, another GPR which signals DHA activity, is highly expressed in the arcuate nucleus of the hypothalamus and the NAc, a structure involved in emotional behavior (Auguste et al., 2016). Interestingly, GPR120 activation by a specific agonist reduces obesity-induced emotional behavior alteration (Auguste et al., 2016). Taken together, these data suggest that several receptors could mediate a direct effect of DHA on neurons to control emotional behavior, opening new avenues in drug development targeting these receptors. However, additional studies are needed to determine whether DHA acts through these receptors to protect from depression and anxiety disorders in humans.

### Endocannabinoid System

**FIGURE 2:**
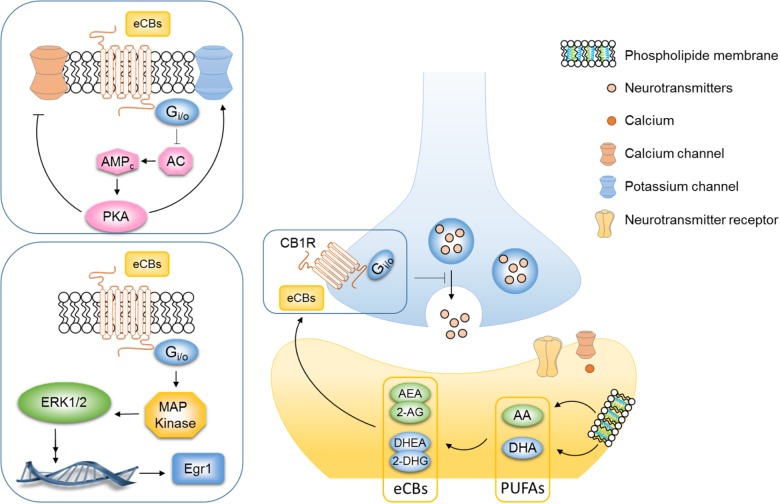
PUFAs are key actors in the regulation of endocannabinoid system. Endocannabinoids are signaling lipids produced from membrane long-chain fatty acid in response to neuronal activity that bind the G-protein-coupled receptor (GPCR) CB1R. The two principal eCBs, AEA and 2-AG, are AA-derived metabolites while DHEA derived from the DHA. eCBs are released into the synaptic cleft and then bind the CB1R on the presynaptic neuron. Activation of CB1R inhibits adenylyl cyclase (AC) activity leading to a subsequent reduction in the cyclic adenosine monophosphate (cAMP) cascade, augmentation of potassium channels, and inhibition of subsequent calcium influx via calcium channels. Consequently, the activation of the CB1R inhibits the release of both excitatory (glutamate) and inhibitory (GABA) neurotransmitters from the presynaptic neuron and decreases synaptic plasticity. The stimulation of CB1R by CB agonists (THC, WIN55,212-2, and CP-55940) or eCBs also activate MAPK signaling pathway. Both eCB-dependent plasticity and CB1R-dependent signaling pathway in brain areas involved in mood-regulation are altered in mice that chronically fed an omega-3 deficient diet.

Regulation of the eCB system could mediate the neuroprotective effect of ω3 PUFAs as both are thought to be involved in depression. The eCB system is in a unique position to link food lipids, neuroplasticity and behavior (Bazinet and Layé, 2014; Bosch-bouju and Layé, 2016; Chianese et al., 2017). eCBs are signaling lipids produced from membrane LC fatty acid in response to neuronal activity and they bind the GPCR CB1R (Mackie, 2008) (**Figure 2**). eCBs are produced on-demand and are rapidly degraded, back into PUFAs or oxidized into active metabolites (Bosch-bouju and Layé, 2016). eCBs include the fatty acid AEA, DHEA, oleylethanolamide and palmitoylethanolamide, as well as 2-AG (Piomelli and Sasso, 2014). The two principal eCBs, AEA and 2-AG, are AA-derived metabolites, while DHEA is derived from the DHA and oleylethanolamide and palmitoylethanolamide is derived from EPA. The most well-studied eCBs are the ω6 PUFA-derived AEA (Devane et al., 1992) and the 2-AG (Sugiura et al., 1995) as compared to the ω3 PUFA-derived eCBs. Activation of CB1 receptors inhibits AC activity leading to a subsequent reduction in the cAMP cascade, augmentation of potassium channels, and inhibition of subsequent calcium influx via calcium channels (**Figure 2**) (Howlett and Fleming, 1984; Howlett, 2002). Consequently, the activation of the CB1R inhibits the release of both excitatory (glutamate) and inhibitory GABA neurotransmitters from presynaptic neurons (Wilson and Nicoll, 2001, 2002; Freund et al., 2003) (**Figure 2**). Finally, numerous important studies have unraveled the key role of eCB system in mood regulation (Morena et al., 2015; Hill and Lee, 2016). As eCBs are derived from ω6 and ω3 PUFA precursors, we hypothesized that the effects of PUFAs on mood-related behavior might be mediated, at least partly, through the eCB system (Lafourcade et al., 2011; Bosch-Bouju et al., 2016; Manduca et al., 2017). As such, inadequate PUFAs ratio during critical time window, i.e., gestation or lactation can lead to changes in eCBs contents in the brain. Newborn piglets that were fed with a diet containing ALA, AA and DHA during the first 18 days of life showed an expected increase of AA and DHA levels in the brain but also of AEA and DHEA metabolites (Berger et al., 2001). Watanabe et al. reported that nutritional ω3 PUFAs deficiency for 2 generations elevates the levels of 2-AG in the mouse brain while ω3 PUFAs supplementation reduces them. In this study, DHA brain levels were affected by dietary ω3 PUFAs deficiency, but not AA the precursor of 2-AG which remained unchanged as compared to the control diet group (Watanabe et al., 2003). It is now well documented that AA levels are barely impacted by PUFAs content of the diet while DHA brain levels are more sensitive to dietary ω6/ω3 PUFAs. Whether increased 2-AG and AEA after exposure to a diet rich in ω6 PUFAs is a compensatory effect to buffer AA concentrations remains to be determined. Lastly a 2-week-supplementation in DHA increased the DHEA and decreased the AEA in brain homogenates in both rats and mice (Wood et al., 2010). One *in vitro* study also demonstrated that unesterified free DHA could directly regulate CB1 gene expression in hippocampal neurons (Pan et al., 2011). Collectively, these reports support the hypothesis proposing that nutritional PUFAs intake is tightly linked to brain eCB levels. By regulating levels of eCBs in the brain, PUFAs have been shown to impact hippocampal synaptic plasticity (Thomazeau et al., 2017) and eCB-dependent plasticity (Lafourcade et al., 2011; Manduca et al., 2017) as well as CB1-associated signaling pathways (Larrieu et al., 2012) in the PFC and NAc. In mice, perinatal exposure to dietary ω3 PUFA deficiency, which leads to low DHA levels in the PFC and the NAc, abolished the eCB-long-term depression in these brain structures. Specifically, this alteration is mediated by an uncoupling from CB1R to its G protein (Lafourcade et al., 2011). Moreover, the effect of the CB1 agonist WIN55,212-2 in anxiety-like behavior was abolished and the CB receptor signaling pathways were altered in the PFC and hypothalamus of ω3 PUFA-deficient mice (Larrieu et al., 2012). A recent study has involved the 2-AG in these aforementioned alterations. Our recent work highlighted that the inhibition of 2-AG degradation normalized emotional behavior deficits and eCB-dependent synaptic plasticity alteration observed in ω3 PUFA-deficient adult mice (Manduca et al., 2017). These observations are the first synaptic and molecular evidence that malnutrition related to ω6/ ω3 PUFAs ratio can have detrimental effect on eCB system, subsequently leading to impaired behavior.

### Hypothalamic–Pituitary–Adrenal Axis

Stress and high trait anxiety are a major risk factor for neuropsychiatric diseases, particularly major depression and anxiety disorders, and are etiologically causal in PTSD (Sandi and Richter-Levin, 2009). Interestingly, although several mechanisms underlying the effects of dietary ω3 PUFA deficiency on emotional behavior have been described (e.g., eCB system), those specifically related to HPA axis function remain poorly understood. Nevertheless, clinical data reported that low plasma DHA levels correlate with higher cerebrospinal fluid CRH levels (Hibbeln et al., 2004) and with higher cortisol in plasma (Nieminen et al., 2006; Mocking et al., 2013). Healthy men receiving supplementation for 3 weeks with dietary fish oil display a blunted cortisol response after an acute mental stress (Delarue et al., 2003). Mood-related deficits observed in deficient mice or rats were recently linked to disrupted GR-mediated signaling pathway, HPA axis hyperactivity as well as eCB system impairment, all involved in mood regulation (Ferraz et al., 2011; Lafourcade et al., 2011; Larrieu et al., 2014, 2015; Bosch-Bouju et al., 2016). Rats that were fed with a ω3 PUFA deficient diet display HPA axis hyper-reactivity after stress exposure reflected by increased levels of plasma corticosterone compared to control diet group (Levant et al., 2008). Conversely, corticosterone hypersecretion induced by a chronic stress and IL-1β exposure is dampened in ω3 PUFA supplemented rats (Song et al., 2003; Ferraz et al., 2011). In Morgese et al. (2016), increased hypothalamic CRF release as well as increased plasmatic corticosterone levels has been shown in ω3 PUFA deficient rats, further demonstrating the link between HPA axis hyperactivity and dietary ω3 PUFAs. In a recent study, we demonstrated that anxiety- and depressive-related behaviors as well as neuronal atrophy in the medial PFC observed in mice fed with a diet deficient in ω3 PUFAs are both mediated by HPA axis hyperactivity (Larrieu et al., 2014). ω3 PUFAs supplementation beyond weaning prevents chronic stress-induced increases in plasma corticosterone levels (Ferraz et al., 2011; Larrieu et al., 2014; Meneses et al., 2017), PFC neuronal shrinkage (Larrieu et al., 2014) as well as anxiety- and depressive-like behaviors (Ferraz et al., 2011; Larrieu et al., 2014). In another study, we confirmed and followed up on their initial observations by demonstrating that GR signaling pathway is compromised in the PFC of ω3 PUFA-deficient mice along with dendritic arborization atrophy (Larrieu et al., 2015). The modulation of neuronal morphology by ω3 PUFAs might be not a generalized phenomenon since neuronal arborization atrophy is only observable in the PFC but not the CA1 of the hippocampus of ω3 PUFA-deficient mice (Delpech et al., 2015b; Larrieu et al., 2015). To further establish the link between dietary ω3 PUFAs consumption and neuronal morphology, *in vitro* studies were conducted showing that PUFAs activate neurites formation and growth in hippocampal (Calderon and Kim, 2004; Cao et al., 2009) and cortical neurons (Cao et al., 2005) in primary culture. Moreover, in these same cultures, a decrease of DHA leads to the reduction of the size of neurites (Ikemoto et al., 1997; Furuya et al., 2002; Calderon and Kim, 2004; Cao et al., 2009). The accretion of DHA in the brain considerably facilitates the formation of the dendritic spines in the hippocampus of Gerbils that were fed with a diet supplemented in DHA (Sakamoto et al., 2007). Interestingly, genetically modified Fat-1 mice that are able to catalyze the conversion of ω6 into ω3 PUFAs display a higher density of spines in the hippocampus compared to WT mice (Kang et al., 2004; He et al., 2009). Moreover, increased spine density in the hippocampus of Fat-1 mice is associated with better cognitive performances assessed in Morris water maze along with increased adult neurogenesis (He et al., 2009). The protective effect of LC ω3 PUFAs could be linked to hippocampal neurogenesis (recently reviewed in (Zainuddin and Thuret, 2012). As such, changes in hippocampal neurogenesis and cell survival in the dentate gyrus have been correlated with depressive-like behavior. A recent study demonstrated that clamping glucocorticoid levels prevent CSDS-induced decreases in neurogenesis and depressive-like behavior in wild type mice, but not in mice with a genetic ablation of neurogenesis (Lehmann et al., 2013). This is particularly relevant knowing that LC ω3 PUFAs supplementation prevents CSDS-induced HPA axis dysregulation (Larrieu et al., 2014). However, whether the beneficial effect of ω3 PUFAs on glucocorticoids and mood is dependent on neurogenesis remains to be evaluated. Finally, an elegant study showed that EPA but not DHA increases neural stem cell proliferation reflected by an increased number of neurospheres bulk via CB1R activity (Dyall et al., 2016). The findings that ω3 PUFAs alone modulate neuronal arborization as well as adult neurogenesis highlight the role of PUFAs as a potent modulator of brain health. Taken together, these studies provide strong validity of nutritional ω3 PUFA-deficient diet as one of the many faces of stress that deeply affects GR-dependent HPA axis function and neuronal morphology plasticity in brain areas associated with emotional behavior.

### Neuroinflammatory Pathways

Inflammation is a key mechanism in the pathophysiology of mood disorders, including major depression, post-partum depression and bipolar disorder (Dantzer et al., 2008; Capuron and Miller, 2011). Increased levels of inflammatory factors, such as proinflammatory cytokines and chemokines, are found in a subset of depressed patients and may contribute to their symptoms through a direct effect in the brain (Raison and Miller, 2011). The mechanisms underlying inflammation and depression have been thoroughly reviewed elsewhere (Capuron and Castanon, 2017). Enhanced peripheral inflammation has also been reported in PTSD (Gill et al., 2008; Newton et al., 2014; Passos et al., 2015; Lerman et al., 2016) and bipolar disorder (Goldstein et al., 2009; Fiedorowicz et al., 2015; Kalelioglu et al., 2015; Uyanik et al., 2015). Importantly, inflammation has been proposed to be key in stress vulnerability and the pathogenesis of major depression (Ménard et al., 2017).

Long chain ω3 PUFAs, DHA, and EPA and their derivatives, so-called SPMs, are well-known regulators of the inflammatory response (Serhan, 2014, 2017). More recently, DHA, EPA, and their derivatives have been shown to also regulate neuroinflammatory processes (Kavanagh et al., 2004; Li et al., 2015; Rey et al., 2016; Dong et al., 2017; Fourrier et al., 2017; Shi et al., 2017; recently reviewed in Layé et al., 2018). Briefly, the expression of the pro-inflammatory cytokine TNFα, IL-6, and IL-1β in the brain (triggered by peripheral or intracerebral administration of LPS, the Gram-negative bacteria endotoxin, amyloid beta administration or associated to aging) is decreased by DHA and EPA dietary supplementation (Labrousse et al., 2012; Orr et al., 2013; Dehkordi et al., 2015; Hopperton et al., 2016). Importantly, in regards to the protective effect of EPA in depression, a dietary supplementation with this fatty acid decreased TNFα expression in the hippocampus following IL-1β central injection (Dong et al., 2017). *In vitro*, DHA, and EPA directly target microglia, the brain innate immune cell (De Smedt-Peyrusse et al., 2008; Antonietta Ajmone-Cat et al., 2012; Pettit et al., 2013; Chang et al., 2015; Fourrier et al., 2017), however, a direct effect of these fatty acids on microglia *in vivo* has not been studied yet. In a model of multiple sclerosis induced by cuprizone, DHA/EPA promote the shift of microglia polarization toward a repair non-inflammatory phenotype (Chen et al., 2014). We have found that the brain content of ω3 PUFA, either increased through the diet or by genetic means, influences microglia and the related neuroinflammatory response to LPS (Mingam et al., 2008; Madore et al., 2014; Delpech et al., 2015a,b; Dinel et al., 2016). In rodent models of neuroinflammation triggered by the intracerebral administration of amyloid-β or cuprizone, brain DHA decreases the number of activated microglia, but not of astrocytes (Hopperton et al., 2016), and promotes an anti-inflammatory phenotype of microglia (Chen et al., 2014). An acute intravenous administration of DHA reduces LPS-induced cytokine production in the hippocampus (Fourrier et al., 2017), but no significant effect of intravenously administered DHA was shown on microglia activation (measured by the upregulation of translocator protein TSPO by Positron-emission tomography) in the injured spinal chord of rat (Tremoleda et al., 2016). DHA and EPA effect on neuroinflammatory pathways could be either direct or indirect. Indeed, LC-PUFAs are converted by COX, LOX, and CYP450 into SPMs, which display pro or anti-inflammatory activities (Chiang and Serhan, 2017), including in the brain (Orr et al., 2013; Rey et al., 2016; Layé et al., 2018). Eicosanoids, resolvins, protectin and maresin derived from DHA and EPA have anti-inflammatory and pro-resolving activities (Bazan, 2009; Serhan et al., 2011). On the opposite, SPMs derived from LA and AA (prostaglandins, leukotrienes or thromboxanes) are mostly pro-inflammatory (Calder, 2006). *In vitro*, DHA derivatives display anti-inflammatory activities in microglia (Marcheselli et al., 2003; Lukiw et al., 2005; Orr et al., 2013; Rey et al., 2016). Brain inflammation triggered by the administration of LPS activates ω6 PUFA derived-prostaglandins production in the brain (Rosenberger et al., 2004; Taha et al., 2017), together with the expression of the enzymes involved in the synthesis of SPMs (Rosenberger et al., 2004; Taha et al., 2017). However, recent work showed that amyloid-β brain infusion, which is proinflammatory, did not increase brain SPMs production (Hopperton et al., 2018). Importantly, PUFAs dietary intervention can modulate cellular levels of both PUFAs and SPMs, with dietary ω6 PUFAs supplementation increasing AA-derived and decreasing EPA-derived SPMs (Taha et al., 2016). Conversely, LC ω3 PUFAs supplementation increasing EPA and DHA-derived SPMs (Balvers et al., 2012; Hashimoto et al., 2015) have not been consistently demonstrated (Hopperton et al., 2018). These observations reinforce the need for more studies to link nutritionnal interventions and SPMs production in specific brain regions.

As previously described, clinical trials using DHA and/or EPA showed mixed results on depressive symptoms. However, based on meta-analysis, EPA has been suggested as a predictor of mood disorder treatment efficiency (Martins, 2009; Sublette et al., 2011; Mocking et al., 2016). Such a positive effect of EPA could be linked to its anti-inflammatory activity. Indeed, in depressed patients, high EPA supplementation is more effective in those with inflammation (Rapaport et al., 2016). In particular, patients with high IL-1 receptor antagonist and C-reactive protein blood levels have greater improvement in mood symptoms in response to EPA, but not DHA enriched dietary supplement. Additional studies with a higher number of patients are warranted to confirm this interesting first study. In addition, whether the higher efficiency of high EPA rather of DHA dietary supplementation is linked to its specific effect on inflammation through specific SPMs remains to be investigated.

## Conclusion and Future Directions

As indicated above, the summarized literature indicate that low ω3 PUFAs intake may predispose certain individuals to depression and anxiety and that dietary supplementation with LC ω3 PUFAs represents an interesting strategy for preventing or treating depression and anxiety disorders in certain individuals. However, several important issues remain to be determined. One of those is the discordant results regarding outcomes in clinical nutritional interventions to investigate the effectiveness of ω3 PUFA supplementation on mood. The unmatched results seem to be partly due to the lack of standardization regarding important parameters such as (i) the inclusion criteria used, (ii) the PUFA composition of the fish oil as well as (iii) the nutritional baseline status of subjects, and (iv) the methods of diagnosis used. We are now beginning to understand how PUFAs affect our brain through a direct sensing effect or an indirect one. This review highlights that ω3 PUFAs, in particular DHA, act onto the brain through a direct effect on FFAR or other indirect mechanisms. We also discussed an indirect effect of ω3 PUFAs on eCB and the HPA axis systems as relevant mechanisms by which dietary ω3 PUFAs modulate mood-related behaviors. Although recent work suggest a causal relationship between nutritional ω3 PUFAs deficiency and alterations of these two systems, major questions remain unanswered, such as how dietary ω3 PUFA maintains HPA axis function to prevent emotional impairment. In this review, we highlighted how powerful dietary PUFAs are in the modulation of the eCB system, which is known to be intimately involved in the regulation of the HPA axis (McLaughlin et al., 2014). As to whether these two mechanisms are interconnected in the effects of ω3 PUFA deficiency-induced depression is yet to be determined. In conclusion, this review reinforces the idea of the usefulness of the dietary ω3 PUFAs as an interesting tool for the design and testing of new non-pharmacological strategies in the treatment of neuropsychiatric disorders such as mood-related disease.

## Author Contributions

All authors listed have made a substantial, direct and intellectual contribution to the work, and approved it for publication.

## Conflict of Interest Statement

The authors declare that the research was conducted in the absence of any commercial or financial relationships that could be construed as a potential conflict of interest.

## Abbreviations

2-AG: 2-arachidonoylglycerol
AA: arachidonic acid
AC: adenylyl cyclase
AEA: ethanolamides anandamide
ALA: alpha-linolenic acid
CA1: cornu ammonis 1
cAMP: cyclic adenosine monophosphate
CB1R: cannabinoid receptor 1
CNS: central nervous system
COX: cyclooxygenase
CRH: corticotrophin-releasing hormone
CSDS: chronic social defeat stress
CYP450: cytochrome P450
DHA: docosahexaenoic acid
DHEA: docosahexaenoyl ethanolamide
DPA: docosapentaenoic acid
eCB: endocannabinoid
EPA: eicosapentaenoic acid
FADS: fatty acid desaturases
FFAR: free fatty acid receptors
FST: forced swimming test
GABA: gamma-aminobutyric acid
GPCR: G-protein-coupled receptor
GPR120: G-protein-coupled receptor 120
GPR40: G-protein-coupled receptor 40
GR: glucocorticoid receptor
HPA: hypothalamic–pituitary–adrenal
IL: interleukin
LA: linoleic acid
LC: long chain
LOX: lipoxygenases
LPS: lipopolysaccharide
NAc: nucleus accumbens
PFC: prefrontal cortex
PPAR: peroxisome proliferator-activated receptor
PTSD: post-traumatic stress disorders
PUFAS: polyunsaturated fatty acids
RXR: retinoid X receptor
SPMs: specialized pro-resolving lipid mediators
SSRI: selective serotonin reuptake inhibitor
TNFα: tumor necrosis factor α
WHO: World Health Organization

## References

[B1] Adams P. B. Lawson S. Sanigorski A. Sinclair A. J. (1996). Arachidonic acid to eicosapentaenoic acid ratio in blood correlates positively with clinical symptoms of depression. *Lipids* 31 S157–S161. 10.1007/BF02637069 8729112

[B2] Aizawa F. Nishinaka T. Yamashita T. Nakamoto K. Kurihara T. Hirasawa A. (2016). GPR40/FFAR1 deficient mice increase noradrenaline levels in the brain and exhibit abnormal behavior. *J. Pharmacol. Sci.* 132 249–254. 10.1016/j.jphs.2016.09.007 27979701

[B3] Antonietta Ajmone-Cat M. Lavinia Salvatori M. De Simone R. Mancini M. Biagioni S. Bernardo A. (2012). Docosahexaenoic acid modulates inflammatory and antineurogenic functions of activated microglial cells. *J. Neurosci. Res.* 90 575–587. 10.1002/jnr.22783 22057807

[B4] Appleton K. M. Peters T. J. Hayward R. C. Heatherley S. V. McNaughton S. A. Rogers P. J. (2007). Depressed mood and n-3 polyunsaturated fatty acid intake from fish: non-linear or confounded association? *Soc. Psychiatry Psychiatr. Epidemiol.* 42 100–104. 10.1007/s00127-006-0142-3 17160592

[B5] Appleton K. M. Rogers P. J. Ness A. R. (2010). Updated systematic review and meta-analysis of the effects of n-3 long-chain polyunsaturated fatty acids on depressed mood. *Am. J. Clin. Nutr.* 91 757–770. 10.3945/ajcn.2009.28313 20130098

[B6] Appleton K. M. Sallis H. M. Perry R. Ness A. R. Churchill R. (2015). Omega-3 fatty acids for depression in adults. *Cochrane Database Syst. Rev.* 5:CD004692. 10.1002/14651858.CD004692.pub4 26537796 PMC5321518

[B7] Auguste S. Fisette A. Fernandes M. F. Hryhorczuk C. Poitout V. Alquier T. (2016). Central agonism of gpr120 acutely inhibits food intake and food reward and chronically suppresses anxiety-like behavior in mice. *Int. J. Neuropsychopharmacol.* 19:pyw014. 10.1093/ijnp/pyw014 26888796 PMC4966276

[B8] Balvers M. G. J. Verhoeckx K. C. M. Bijlsma S. Rubingh C. M. Meijerink J. Wortelboer H. M. (2012). Fish oil and inflammatory status alter the n-3 to n-6 balance of the endocannabinoid and oxylipin metabolomes in mouse plasma and tissues. *Metabolomics* 8 1130–1147. 10.1007/s11306-012-0421-9 23136559 PMC3483099

[B9] Barberger-Gateau P. Jutand M. A. Letenneur L. Larrieu S. Tavernier B. Berr C. (2005). Correlates of regular fish consumption in French elderly community dwellers: data from the three-City study. *Eur. J. Clin. Nutr.* 59 817–825. 10.1038/sj.ejcn.1602145 15900310

[B10] Bazan N. G. (2009). Neuroprotectin D1-mediated anti-inflammatory and survival signaling in stroke, retinal degenerations, and Alzheimer’s disease. *J. Lipid Res.* 50(Suppl.), S400–S405. 10.1194/jlr.R800068-JLR200 19018037 PMC2674685

[B11] Bazinet R. P. Layé S. (2014). Polyunsaturated fatty acids and their metabolites in brain function and disease. *Nat. Rev. Neurosci.* 15 771–785. 10.1038/nrn3820 25387473

[B12] Berger A. Crozier G. Bisogno T. Cavaliere P. Innis S. Di Marzo V. (2001). Anandamide and diet: inclusion of dietary arachidonate and docosahexaenoate leads to increased brain levels of the corresponding N-acylethanolamines in piglets. *Proc. Natl. Acad. Sci. U.S.A.* 98 6402–6406. 10.1073/pnas.101119098 11353819 PMC33480

[B13] Berton O. McClung C. A. Dileone R. J. Krishnan V. Renthal W. Russo S. J. (2006). Essential role of BDNF in the mesolimbic dopamine pathway in social defeat stress. *Science* 311 864–868. 10.1126/science.1120972 16469931

[B14] Bondi C. O. Taha A. Y. Tock J. L. Totah N. K. B. Cheon Y. Torres G. E. (2014). Adolescent behavior and dopamine availability are uniquely sensitive to dietary omega-3 fatty acid deficiency. *Biol. Psychiatry* 75 38–46. 10.1016/j.biopsych.2013.06.007 23890734 PMC3858419

[B15] Bosch-Bouju C. Larrieu T. Linders L. Manzoni O. J. Layé S. (2016). Endocannabinoid-mediated plasticity in nucleus accumbens controls vulnerability to anxiety after social defeat stress. *Cell Rep.* 16 1237–1242. 10.1016/j.celrep.2016.06.082 27452462

[B16] Bosch-bouju C. Layé S. (2016). *Dietary Omega-6 / Omega-3 and Endocannabinoids: Implications for Brain Health and Diseases*. Osaka: InTech 10.5772/62498

[B17] Bozzatello P. Brignolo E. De Grandi E. Bellino S. (2016). Supplementation with omega-3 fatty acids in psychiatric disorders: a review of literature data. *J. Clin. Med.* 5:E67. 10.3390/jcm5080067 27472373 PMC4999787

[B18] Bradbury J. (2011). Docosahexaenoic acid (DHA): an ancient nutrient for the modern human brain. *Nutrients* 3 529–554. 10.3390/nu3050529 22254110 PMC3257695

[B19] Bradbury J. Myers S. P. Meyer B. Brooks L. Peake J. Sinclair A. J. (2017). Chronic psychological stress was not ameliorated by omega-3 eicosapentaenoic acid (EPA). *Front. Pharmacol.* 8:551 10.3389/fphar.2017.00551 PMC5671493 29163147

[B20] Brunoni A. R. Fraguas R. Fregni F. (2009). Pharmacological and combined interventions for the acute depressive episode: focus on efficacy and tolerability. *Ther. Clin. Risk Manag.* 5 897–910. 19956554 10.2147/tcrm.s5751 PMC2781064

[B21] Burdge G. C. Finnegan Y. E. Minihane A. M. Williams C. M. Wootton S. A. (2003). Effect of altered dietary n-3 fatty acid intake upon plasma lipid fatty acid composition, conversion of [13C]alpha-linolenic acid to longer-chain fatty acids and partitioning towards beta-oxidation in older men. *Br. J. Nutr.* 90 311–321. 10.1079/BJN2003901 12908891

[B22] Burr G. O. Burr M. M. (1929). A new deficiency disease produced by the rigid exclusion of fat from the diet. *J. Biol. Chem.* 82 345–367. 4586201 10.1111/j.1753-4887.1973.tb06008.x

[B23] Calder P. C. (2006). Polyunsaturated fatty acids and inflammation. *Prostaglandins Leukot. Essent. Fatty Acids* 75 197–202. 10.1016/j.plefa.2006.05.012 16828270

[B24] Calderon F. Kim H.-Y. (2004). Docosahexaenoic acid promotes neurite growth in hippocampal neurons. *J. Neurochem.* 90 979–988. 10.1111/j.1471-4159.2004.02520.x 15287904

[B25] Calderon F. Kim H.-Y. (2007). Role of RXR in neurite outgrowth induced by docosahexaenoic acid. *Prostaglandins. Leukot. Essent. Fatty Acids* 77 227–232. 10.1016/j.plefa.2007.10.026 18036800 PMC2174793

[B26] Cao D. Kevala K. Kim J. Moon H.-S. Jun S. B. Lovinger D. (2009). Docosahexaenoic acid promotes hippocampal neuronal development and synaptic function. *J. Neurochem.* 111 510–521. 10.1111/j.1471-4159.2009.06335.x 19682204 PMC2773444

[B27] Cao D. Xue R. Xu J. Liu Z. (2005). Effects of docosahexaenoic acid on the survival and neurite outgrowth of rat cortical neurons in primary cultures. *J. Nutr. Biochem.* 16 538–546. 10.1016/j.jnutbio.2005.02.002 16115542

[B28] Capuron L. Castanon N. (2017). Role of inflammation in the development of neuropsychiatric symptom domains: evidence and mechanisms. *Curr. Top. Behav. Neurosci.* 31 31–44. 10.1007/7854_2016_14 27221626

[B29] Capuron L. Miller A. H. (2011). Immune system to brain signaling: neuropsychopharmacological implications. *Pharmacol. Ther.* 130 226–238. 10.1016/j.pharmthera.2011.01.014 21334376 PMC3072299

[B30] Carlezon W. A. Mague S. D. Parow A. M. Stoll A. L. Cohen B. M. Renshaw P. F. (2005). Antidepressant-like effects of uridine and omega-3 fatty acids are potentiated by combined treatment in rats. *Biol. Psychiatry* 57 343–350. 10.1016/j.biopsych.2004.11.038 15705349

[B31] Carney R. M. Steinmeyer B. C. Freedland K. E. Rubin E. H. Rich M. W. Harris W. S. (2016). Baseline blood levels of omega-3 and depression remission. *J. Clin. Psychiatry* 77 e138–e143. 10.4088/JCP.14m09660 26930527 PMC5369023

[B32] Carrié I. Clément M. de Javel D. Francès H. Bourre J. M. (2000a). Phospholipid supplementation reverses behavioral and biochemical alterations induced by n-3 polyunsaturated fatty acid deficiency in mice. *J. Lipid Res.* 41 473–480. 10706595

[B33] Carrié I. Clément M. de Javel D. Francès H. Bourre J. M. (2000b). Specific phospholipid fatty acid composition of brain regions in mice. Effects of n-3 polyunsaturated fatty acid deficiency and phospholipid supplementation. *J. Lipid Res.* 41 465–472. 10706594

[B34] Casali B. T. Corona A. W. Mariani M. M. Karlo J. C. Ghosal K. Landreth G. E. (2015). Omega-3 fatty acids augment the actions of nuclear receptor agonists in a mouse model of Alzheimer’s Disease. *J. Neurosci.* 35 9173–9181. 10.1523/JNEUROSCI.1000-15.2015 26085639 PMC4469742

[B35] Chang P. K.-Y. Khatchadourian A. McKinney R. A. Maysinger D. (2015). Docosahexaenoic acid (DHA): a modulator of microglia activity and dendritic spine morphology. *J. Neuroinflamm.* 12:34. 10.1186/s12974-015-0244-245 25889069 PMC4344754

[B36] Chen C. T. Bazinet R. P. (2015). β-oxidation and rapid metabolism, but not uptake regulate brain eicosapentaenoic acid levels. *Prostaglandins Leukot. Essent. Fatty Acids* 92 33–40. 10.1016/j.plefa.2014.05.007 24986271

[B37] Chen S. Zhang H. Pu H. Wang G. Li W. Leak R. K. (2014). n-3 PUFA supplementation benefits microglial responses to myelin pathology. *Sci. Rep.* 4:7458. 10.1038/srep07458 25500548 PMC4264015

[B38] Chianese R. Coccurello R. Viggiano A. Scafuro M. Fiore M. Coppola G. (2017). Impact of dietary fats on brain functions. *Curr. Neuropharmacol.* 10.2174/1570159X15666171017102547 [Epub ahead of print]. 29046155 PMC6120115

[B39] Chiang N. Serhan C. N. (2017). Structural elucidation and physiologic functions of specialized pro-resolving mediators and their receptors. *Mol. Aspects Med.* 58 114–129. 10.1016/j.mam.2017.03.005 28336292 PMC5623601

[B40] Clouard C. Souza A. S. Gerrits W. J. Hovenier R. Lammers A. Bolhuis J. E. (2015). Maternal fish oil supplementation affects the social behavior, brain fatty acid profile, and sickness response of piglets. *J. Nutr.* 145 2176–2184. 10.3945/jn.115.214650 26180250

[B41] Colangelo L. A. He K. Whooley M. A. Daviglus M. L. Liu K. (2009). Higher dietary intake of long-chain omega-3 polyunsaturated fatty acids is inversely associated with depressive symptoms in women. *Nutrition* 25 1011–1019. 10.1016/j.nut.2008.12.008 19195841 PMC2798585

[B42] Coulombe K. Kerdiles O. Tremblay C. Emond V. Lebel M. Boulianne A.-S. (2017). Impact of DHA intake in a mouse model of synucleinopathy. *Exp. Neurol.* 301 39–49. 10.1016/j.expneurol.2017.12.002 29229294

[B43] Cribb L. Murphy J. Froud A. Oliver G. Bousman C. A. Ng C. H. (2017). Erythrocyte polyunsaturated fatty acid composition is associated with depression and FADS genotype in Caucasians. *Nutr. Neurosci.* 10.1080/1028415X.2017.1327685 [Epub ahead of print]. 28552045

[B44] Cunnane S. C. (2003). Problems with essential fatty acids: time for a new paradigm? *Prog. Lipid Res.* 42 544–568. 10.1016/S0163-7827(03)00038-9 14559071

[B45] Dantzer R. O’Connor J. C. Freund G. G. Johnson R. W. Kelley K. W. (2008). From inflammation to sickness and depression: when the immune system subjugates the brain. *Nat. Rev. Neurosci.* 9 46–56. 10.1038/nrn2297 18073775 PMC2919277

[B46] de la Presa Owens S. Innis S. M. (1999). Docosahexaenoic and arachidonic acid prevent a decrease in dopaminergic and serotoninergic neurotransmitters in frontal cortex caused by a linoleic and alpha-linolenic acid deficient diet in formula-fed piglets. *J. Nutr.* 129 2088–2093. 10.1093/jn/129.11.2088 10539789

[B47] De Smedt-Peyrusse V. Sargueil F. Moranis A. Harizi H. Mongrand S. Layé S. (2008). Docosahexaenoic acid prevents lipopolysaccharide-induced cytokine production in microglial cells by inhibiting lipopolysaccharide receptor presentation but not its membrane subdomain localization. *J. Neurochem.* 105 296–307. 10.1111/j.1471-4159.2007.05129.x 18021297

[B48] de Vries G.-J. Mocking R. Lok A. Assies J. Schene A. Olff M. (2016). Fatty acid concentrations in patients with posttraumatic stress disorder compared to healthy controls. *J. Affect. Disord.* 205 351–359. 10.1016/j.jad.2016.08.021 27567082

[B49] Dehkordi N. G. Noorbakhshnia M. Ghaedi K. Esmaeili A. Dabaghi M. (2015). Omega-3 fatty acids prevent LPS-induced passive avoidance learning and memory and CaMKII-α gene expression impairments in hippocampus of rat. *Pharmacol. Rep.* 67 370–375. 10.1016/j.pharep.2014.10.014 25712666

[B50] Delarue J. Matzinger O. Binnert C. Schneiter P. Chioléro R. Tappy L. (2003). Fish oil prevents the adrenal activation elicited by mental stress in healthy men. *Diabetes Metab.* 29 289–295. 10.1016/S1262-3636(07)70039-3 12909818

[B51] Delion S. Chalon S. Hérault J. Guilloteau D. Besnard J. C. Durand G. (1994). Chronic dietary alpha-linolenic acid deficiency alters dopaminergic and serotoninergic neurotransmission in rats. *J. Nutr.* 124 2466–2476. 10.1093/jn/124.12.2466 16856329

[B52] Delpech J.-C. Madore C. Joffre C. Aubert A. Kang J. X. Nadjar A. (2015a). Transgenic increase in n-3/n-6 fatty acid ratio protects against cognitive deficits induced by an immune challenge through decrease of neuroinflammation. *Neuropsychopharmacology* 40 525–536. 10.1038/npp.2014.196 25228141 PMC4289942

[B53] Delpech J.-C. Thomazeau A. Madore C. Bosch-Bouju C. Larrieu T. Lacabanne C. (2015b). Dietary n-3 PUFAs deficiency increases vulnerability to inflammation-induced spatial memory impairment. *Neuropsychopharmacology* 40 2774–2787. 10.1038/npp.2015.127 25948102 PMC4864653

[B54] DeMar J. C. Ma K. Bell J. M. Igarashi M. Greenstein D. Rapoport S. I. (2006). One generation of n-3 polyunsaturated fatty acid deprivation increases depression and aggression test scores in rats. *J. Lipid Res.* 47 172–180. 10.1194/jlr.M500362-JLR200 16210728

[B55] Devane W. A. Hanus L. Breuer A. Pertwee R. G. Stevenson L. A. Griffin G. (1992). Isolation and structure of a brain constituent that binds to the cannabinoid receptor. *Science* 258 1946–1949. 10.1126/science.1470919 1470919

[B56] Dinel A. L. Rey C. Baudry C. Fressange-Mazda C. Le Ruyet P. Nadjar A. (2016). Enriched dairy fat matrix diet prevents early life lipopolysaccharide-induced spatial memory impairment at adulthood. *Prostaglandins Leukot. Essent. Fatty Acids* 113 9–18. 10.1016/j.plefa.2016.08.013 27720041

[B57] Dong Y. Xu M. Kalueff A. V. Song C. (2017). Dietary eicosapentaenoic acid normalizes hippocampal omega-3 and 6 polyunsaturated fatty acid profile, attenuates glial activation and regulates BDNF function in a rodent model of neuroinflammation induced by central interleukin-1β administration. *Eur. J. Nutr.* 57 1781–1791. 10.1007/s00394-017-1462-7 28523372

[B58] Dragano N. R. V. Solon C. Ramalho A. F. de Moura R. F. Razolli D. S. Christiansen E. (2017). Polyunsaturated fatty acid receptors, GPR40 and GPR 120, are expressed in the hypothalamus and control energy homeostasis and inflammation. *J. Neuroinflammation* 14:91. 10.1186/s12974-017-0869-7 28446241 PMC5405534

[B59] Dyall S. C. Mandhair H. K. Fincham R. E. A. Kerr D. M. Roche M. Molina-Holgado F. (2016). Distinctive effects of eicosapentaenoic and docosahexaenoic acids in regulating neural stem cell fate are mediated via endocannabinoid signalling pathways. *Neuropharmacology* 107 387–395. 10.1016/j.neuropharm.2016.03.055 27044662

[B60] Edwards R. Peet M. Shay J. Horrobin D. (1998a). Depletion of docosahexaenoic acid in red blood cell membranes of depressive patients. *Biochem. Soc. Trans.* 26:S142 10.1042/bst026s142 9649817

[B61] Edwards R. Peet M. Shay J. Horrobin D. (1998b). Omega-3 polyunsaturated fatty acid levels in the diet and in red blood cell membranes of depressed patients. *J. Affect. Disord.* 48 149–155. 9543204 10.1016/s0165-0327(97)00166-3

[B62] Emken E. A. Adlof R. O. Gulley R. M. (1994). Dietary linoleic acid influences desaturation and acylation of deuterium-labeled linoleic and linolenic acids in young adult males. *Biochim. Biophys. Acta* 1213 277–288. 10.1016/0005-2760(94)00054-9 7914092

[B63] Fallin M. D. Lasseter V. K. Wolyniec P. S. McGrath J. A. Nestadt G. Valle D. (2004). Genomewide linkage scan for bipolar-disorder susceptibility loci among Ashkenazi Jewish families. *Am. J. Hum. Genet.* 75 204–219. 10.1086/422474 15208783 PMC1216055

[B64] Favrelière S. Barrier L. Durand G. Chalon S. Tallineau C. (1998). Chronic dietary n-3 polyunsaturated fatty acids deficiency affects the fatty acid composition of plasmenylethanolamine and phosphatidylethanolamine differently in rat frontal cortex, striatum, and cerebellum. *Lipids* 33 401–407. 10.1007/s11745-998-0221-y 9590628

[B65] Fedorova I. Salem N. (2006). Omega-3 fatty acids and rodent behavior. *Prostaglandins Leukot. Essent. Fatty Acids* 75 271–289. 10.1016/j.plefa.2006.07.006 16973342

[B66] Fernandes P. G. Cook R. M. (2013). Reversal of fish stock decline in the Northeast Atlantic. *Curr. Biol.* 23 1432–1437. 10.1016/j.cub.2013.06.016 23871238

[B67] Ferraz A. C. Delattre A. M. Almendra R. G. Sonagli M. Borges C. Araujo P. (2011). Chronic n -3 fatty acids supplementation promotes beneficial effects on anxiety, cognitive and depressive-like behaviors in rats subjected to a restraint stress protocol. *Behav. Brain Res.* 219 116–122. 10.1016/j.bbr.2010.12.028 21192985

[B68] Fiedorowicz J. G. Prossin A. R. Johnson C. P. Christensen G. E. Magnotta V. A. Wemmie J. A. (2015). Peripheral inflammation during abnormal mood states in bipolar I disorder. *J. Affect. Disord.* 187 172–178. 10.1016/j.jad.2015.08.036 26339927 PMC4587340

[B69] Fourrier C. Remus-Borel J. Greenhalgh A. D. Guichardant M. Bernoud-Hubac N. Lagarde M. (2017). Docosahexaenoic acid-containing choline phospholipid modulates LPS-induced neuroinflammation in vivo and in microglia in vitro. *J. Neuroinflammation* 14:170. 10.1186/s12974-017-0939-x 28838312 PMC5571638

[B70] Francès H. Monier C. Bourre J. M. (1995). Effects of dietary alpha-linolenic acid deficiency on neuromuscular and cognitive functions in mice. *Life Sci.* 57 1935–1947. 10.1016/0024-3205(95)02180-Q 7475943

[B71] Frasure-Smith N. Lespérance F. Julien P. (2004). Major depression is associated with lower omega-3 fatty acid levels in patients with recent acute coronary syndromes. *Biol. Psychiatry* 55 891–896. 10.1016/j.biopsych.2004.01.021 15110732

[B72] Freeman M. P. Davis M. Sinha P. Wisner K. L. Hibbeln J. R. Gelenberg A. J. (2008). Omega-3 fatty acids and supportive psychotherapy for perinatal depression: a randomized placebo-controlled study. *J. Affect. Disord.* 110 142–148. 10.1016/j.jad.2007.12.228 18206247 PMC5598081

[B73] Freund T. F. Katona I. Piomelli D. (2003). Role of endogenous cannabinoids in synaptic signaling. *Physiol. Rev.* 83 1017–1066. 10.1152/physrev.00004.2003 12843414

[B74] Furuya H. Watanabe T. Sugioka Y. Inagaki Y. Okazaki I. (2002). Effect of ethanol and docosahexaenoic acid on nerve growth factor-induced neurite formation and neuron specific growth-associated protein gene expression in PC12 cells. *Nihon Arukoru Yakubutsu Igakkai Zasshi* 37 513–522. 12462067

[B75] Ganança L. Galfalvy H. C. Oquendo M. A. Hezghia A. Cooper T. B. Mann J. J. (2017). Lipid correlates of antidepressant response to omega-3 polyunsaturated fatty acid supplementation: a pilot study. *Prostaglandins Leukot. Essent. Fat. Acids* 119 38–44. 10.1016/j.plefa.2017.03.004 28410668 PMC5487266

[B76] Gertsik L. Poland R. E. Bresee C. Rapaport M. H. (2012). Omega-3 fatty acid augmentation of citalopram treatment for patients with major depressive disorder. *J. Clin. Psychopharmacol.* 32 61–64. 10.1097/JCP.0b013e31823f3b5f 22198441 PMC3375825

[B77] Gibson R. A. Makrides M. (2001). Long-chain polyunsaturated fatty acids in breast milk: are they essential? *Adv. Exp. Med. Biol.* 501 375–383. 10.1007/978-1-4615-1371-1_46 11787705

[B78] Gibson R. A. Muhlhausler B. Makrides M. (2011). Conversion of linoleic acid and alpha-linolenic acid to long-chain polyunsaturated fatty acids (LCPUFAs), with a focus on pregnancy, lactation and the first 2 years of life. *Matern. Child Nutr.* 7(Suppl. 2), 17–26. 10.1111/j.1740-8709.2011.00299.x 21366864 PMC6860743

[B79] Gill J. Vythilingam M. Page G. G. (2008). Low cortisol, high DHEA, and high levels of stimulated TNF-alpha, and IL-6 in women with PTSD. *J. Trauma. Stress* 21 530–539. 10.1002/jts.20372 19107725 PMC2829297

[B80] Golden S. A. Covington H. E. Berton O. Russo S. J. (2011). A standardized protocol for repeated social defeat stress in mice. *Nat. Protoc.* 6 1183–1191. 10.1038/nprot.2011.361 21799487 PMC3220278

[B81] Goldstein B. I. Kemp D. E. Soczynska J. K. McIntyre R. S. (2009). Inflammation and the phenomenology, pathophysiology, comorbidity, and treatment of bipolar disorder: a systematic review of the literature. *J. Clin. Psychiatry* 70 1078–1090. 10.4088/JCP.08r04505 19497250

[B82] Goyens P. L. L. Spilker M. E. Zock P. L. Katan M. B. Mensink R. P. (2006). Conversion of alpha-linolenic acid in humans is influenced by the absolute amounts of alpha-linolenic acid and linoleic acid in the diet and not by their ratio. *Am. J. Clin. Nutr.* 84 44–53. 10.1093/ajcn/84.1.44 16825680

[B83] Green P. Hermesh H. Monselise A. Marom S. Presburger G. Weizman A. (2006). Red cell membrane omega-3 fatty acids are decreased in nondepressed patients with social anxiety disorder. *Eur. Neuropsychopharmacol.* 16 107–113. 10.1016/j.euroneuro.2005.07.005 16243493

[B84] Grosso G. Pajak A. Marventano S. Castellano S. Galvano F. Bucolo C. (2014). Role of omega-3 fatty acids in the treatment of depressive disorders: a comprehensive meta-analysis of randomized clinical trials. *PLoS One* 9:e96905. 10.1371/journal.pone.0096905 24805797 PMC4013121

[B85] Hallahan B. Ryan T. Hibbeln J. R. Murray I. T. Glynn S. Ramsden C. E. (2016). Efficacy of omega-3 highly unsaturated fatty acids in the treatment of depression. *Br. J. Psychiatry* 209 192–201. 10.1192/bjp.bp.114.160242 27103682 PMC9406129

[B86] Hashimoto M. Katakura M. Tanabe Y. Al Mamun A. Inoue T. Hossain S. (2015). n-3 fatty acids effectively improve the reference memory-related learning ability associated with increased brain docosahexaenoic acid-derived docosanoids in aged rats. *Biochim. Biophys. Acta* 1851 203–209. 10.1016/j.bbalip.2014.10.009 25450447

[B87] He C. Qu X. Cui L. Wang J. Kang J. X. (2009). Improved spatial learning performance of fat-1 mice is associated with enhanced neurogenesis and neuritogenesis by docosahexaenoic acid. *Proc. Natl. Acad. Sci. U.S.A.* 106 11370–11375. 10.1073/pnas.0904835106 19549874 PMC2708766

[B88] Hibbeln J. R. (1998). Fish consumption and major depression. *Lancet* 351:1213 10.1016/S0140-6736(05)79168-6 9643729

[B89] Hibbeln J. R. (2002). Seafood consumption, the DHA content of mothers’ milk and prevalence rates of postpartum depression: a cross-national, ecological analysis. *J. Affect. Disord.* 69 15–29. 10.1016/S0165-0327(01)00374-3 12103448

[B90] Hibbeln J. R. Bissette G. Umhau J. C. George D. T. (2004). Omega-3 status and cerebrospinal fluid corticotrophin releasing hormone in perpetrators of domestic violence. *Biol. Psychiatry* 56 895–897. 10.1016/j.biopsych.2004.08.021 15576068

[B91] Hibbeln J. R. Salem N. (1995). Dietary polyunsaturated fatty acids and depression: when cholesterol does not satisfy. *Am. J. Clin. Nutr.* 62 1–9. 10.1093/ajcn/62.1.1 7598049

[B92] Hill M. N. Lee F. S. (2016). Endocannabinoids and stress resilience: is deficiency sufficient to promote vulnerability? *Biol. Psychiatry* 79 792–793. 10.1016/j.biopsych.2016.03.2099 27130852 PMC5086031

[B93] Hirasawa A. Tsumaya K. Awaji T. Katsuma S. Adachi T. Yamada M. (2005). Free fatty acids regulate gut incretin glucagon-like peptide-1 secretion through GPR120. *Nat. Med.* 11 90–94. 10.1038/nm1168 15619630

[B94] Hopperton K. E. Trépanier M.-O. Giuliano V. Bazinet R. P. (2016). Brain omega-3 polyunsaturated fatty acids modulate microglia cell number and morphology in response to intracerebroventricular amyloid-β 1-40 in mice. *J. Neuroinflammation* 13:257. 10.1186/s12974-016-0721-5 27688126 PMC5041295

[B95] Hopperton K. E. Trépanier M.-O. James N. C. E. Chouinard-Watkins R. Bazinet R. P. (2018). Fish oil feeding attenuates neuroinflammatory gene expression without concomitant changes in brain eicosanoids and docosanoids in a mouse model of Alzheimer’s disease. *Brain Behav. Immun.* 69 74–90. 10.1016/j.bbi.2017.11.002 29109025

[B96] Howlett A. C. (2002). The cannabinoid receptors. *Prostaglandins Other Lipid Mediat.* 68–69, 619–631. 10.1016/S0090-6980(02)00060-6 12432948

[B97] Howlett A. C. Fleming R. M. (1984). Cannabinoid inhibition of adenylate cyclase. Pharmacology of the response in neuroblastoma cell membranes. *Mol. Pharmacol.* 26 532–538. 6092901

[B98] Huang S.-Y. Yang H.-T. Chiu C.-C. Pariante C. M. Su K.-P. (2008). Omega-3 fatty acids on the forced-swimming test. *J. Psychiatr. Res.* 42 58–63. 10.1016/j.jpsychires.2006.09.004 17070845

[B99] Ikemoto A. Kobayashi T. Watanabe S. Okuyama H. (1997). Membrane fatty acid modifications of PC12 cells by arachidonate or docosahexaenoate affect neurite outgrowth but not norepinephrine release. *Neurochem. Res.* 22 671–678. 10.1023/A:1027393724676 9178949

[B100] Innis S. M. (2007). Dietary (n-3) fatty acids and brain development. *J. Nutr.* 137 855–859. 10.1093/jn/137.4.855 17374644

[B101] Itoh Y. Kawamata Y. Harada M. Kobayashi M. Fujii R. Fukusumi S. (2003). Free fatty acids regulate insulin secretion from pancreatic beta cells through GPR40. *Nature* 422 173–176. 10.1038/nature01478 12629551

[B102] Jazayeri S. Tehrani-Doost M. Keshavarz S. A. Hosseini M. Djazayery A. Amini H. (2008). Comparison of therapeutic effects of omega-3 fatty acid eicosapentaenoic acid and fluoxetine, separately and in combination, in major depressive disorder. *Aust. N. Z. J. Psychiatry* 42 192–198. 10.1080/00048670701827275 18247193

[B103] Jensen H. A. R. Harsløf L. B. S. Nielsen M. S. Christensen L. B. Ritz C. Michaelsen K. F. (2014). FADS single-nucleotide polymorphisms are associated with behavioral outcomes in children, and the effect varies between sexes and is dependent on PPAR genotype. *Am. J. Clin. Nutr.* 100 826–832. 10.3945/ajcn.114.087882 25080457

[B104] Joffre C. Nadjar A. Lebbadi M. Calon F. Laye S. (2014). n-3 LCPUFA improves cognition: the young, the old and the sick. *Prostaglandins Leukot. Essent. Fatty Acids* 91 1–20. 10.1016/j.plefa.2014.05.001 24908517

[B105] Kalelioglu T. Akkus M. Karamustafalioglu N. Genc A. Genc E. S. Cansiz A. (2015). Neutrophil-lymphocyte and platelet-lymphocyte ratios as inflammation markers for bipolar disorder. *Psychiatry Res.* 228 925–927. 10.1016/j.psychres.2015.05.110 26154814

[B106] Kamphuis M. H. Geerlings M. I. Tijhuis M. A. R. Kalmijn S. Grobbee D. E. Kromhout D. (2006). Depression and cardiovascular mortality: a role for n-3 fatty acids? *Am. J. Clin. Nutr.* 84 1513–1517. 10.1093/ajcn/84.6.1513 17158437

[B107] Kang J. X. Wang J. Wu L. Kang Z. B. (2004). Transgenic mice: fat-1 mice convert n-6 to n-3 fatty acids. *Nature* 427:504. 10.1038/427504a 14765186

[B108] Kavanagh T. Lonergan P. E. Lynch M. A. (2004). Eicosapentaenoic acid and gamma-linolenic acid increase hippocampal concentrations of IL-4 and IL-10 and abrogate lipopolysaccharide-induced inhibition of long-term potentiation. *Prostaglandins Leukot. Essent. Fatty Acids* 70 391–397. 10.1016/j.plefa.2003.12.014 15041032

[B109] Kiecolt-Glaser J. K. Belury M. A. Andridge R. Malarkey W. B. Glaser R. (2011). Omega-3 supplementation lowers inflammation and anxiety in medical students: a randomized controlled trial. *Brain Behav. Immun.* 25 1725–1734. 10.1016/j.bbi.2011.07.229 21784145 PMC3191260

[B110] Koletzko B. Lattka E. Zeilinger S. Illig T. Steer C. (2011). Genetic variants of the fatty acid desaturase gene cluster predict amounts of red blood cell docosahexaenoic and other polyunsaturated fatty acids in pregnant women: findings from the Avon Longitudinal Study of Parents and Children. *Am. J. Clin. Nutr.* 93 211–219. 10.3945/ajcn.110.006189 21106917

[B111] Kraguljac N. V. Montori V. M. Pavuluri M. Chai H. S. Wilson B. S. Unal S. S. (2009). Efficacy of omega-3 fatty acids in mood disorders - a systematic review and metaanalysis. *Psychopharmacol. Bull.* 42 39–54. 19752840

[B112] Krishnan V. Han M.-H. Graham D. L. Berton O. Renthal W. Russo S. J. (2007). Molecular adaptations underlying susceptibility and resistance to social defeat in brain reward regions. *Cell* 131 391–404. 10.1016/j.cell.2007.09.018 17956738

[B113] Krzyzosiak A. Szyszka-Niagolov M. Wietrzych M. Gobaille S. Muramatsu S. Krezel W. (2010). Retinoid x receptor gamma control of affective behaviors involves dopaminergic signaling in mice. *Neuron* 66 908–920. 10.1016/j.neuron.2010.05.004 20620876

[B114] Labrousse V. F. Nadjar A. Joffre C. Costes L. Aubert A. Grégoire S. (2012). Short-term long chain omega3 diet protects from neuroinflammatory processes and memory impairment in aged mice. *PLoS One* 7:e36861. 10.1371/journal.pone.0036861 22662127 PMC3360741

[B115] Lacombe R. J. S. Chouinard-Watkins R. Bazinet R. P. (2018). Brain docosahexaenoic acid uptake and metabolism. *Mol. Aspects. Med.* 10.1016/j.mam.2017.12.004 [Epub ahead of print]. 29305120

[B116] Lafourcade M. Larrieu T. Mato S. Duffaud A. Sepers M. Matias I. (2011). Nutritional omega-3 deficiency abolishes endocannabinoid-mediated neuronal functions. *Nat. Neurosci.* 14 345–350. 10.1038/nn.2736 21278728

[B117] Larrieu T. Cherix A. Duque A. Rodrigues J. Lei H. Gruetter R. (2017). Hierarchical status predicts behavioral vulnerability and nucleus accumbens metabolic profile following chronic social defeat stress. *Curr. Biol.* 27 2202.e4–2210.e4. 10.1016/j.cub.2017.06.027 28712571

[B118] Larrieu T. Hilal M. L. Desmedt-peyrusse V. Sans N. Layé S. (2015). Nutritional omega-3 deficiency alters glucocorticoid receptor-signaling pathway and neuronal morphology in regionally distinct brain structures associated with emotional deficits. *Neural Plast.* 2016:8574830. 10.1155/2016/8574830 27057368 PMC4710953

[B119] Larrieu T. Hilal M. L. Fourrier C. Desmedt-Peyrusse V. Sans N. Capuron L. (2014). Nutritional omega-3 modulates neuronal morphology in the prefrontal cortex along with depression-related behaviour through corticosterone secretion. *Transl. Psychiatry* 4:e437. 10.1038/tp.2014.77 25203168 PMC4203007

[B120] Larrieu T. Madore C. Joffre C. Layé S. (2012). Nutritional n-3 polyunsaturated fatty acids deficiency alters cannabinoid receptor signaling pathway in the brain and associated anxiety-like behavior in mice. *J. Physiol. Biochem.* 68 671–681. 10.1007/s13105-012-0179-6 22707188

[B121] Layé S. Nadjar A. Joffre C. Bazinet R. P. (2018). Anti-inflammatory effects of omega-3 fatty acids in the brain: physiological mechanisms and relevance to pharmacology. *Pharmacol. Rev.* 70 12–38. 10.1124/pr.117.014092 29217656

[B122] Lehmann M. L. Brachman R. A. Martinowich K. Schloesser R. J. Herkenham M. (2013). Glucocorticoids orchestrate divergent effects on mood through adult neurogenesis. *J. Neurosci.* 33 2961–2972. 10.1523/JNEUROSCI.3878-12.2013 23407954 PMC3711562

[B123] Lemaitre R. N. Siscovick D. S. Berry E. M. Kark J. D. Friedlander Y. (2008). Familial aggregation of red blood cell membrane fatty acid composition: the kibbutzim family study. *Metabolism* 57 662–668. 10.1016/j.metabol.2007.12.011 18442630

[B124] Lengqvist J. Mata De Urquiza A. Bergman A.-C. Willson T. M. Sjövall J. Perlmann T. (2004). Polyunsaturated fatty acids including docosahexaenoic and arachidonic acid bind to the retinoid X receptor alpha ligand-binding domain. *Mol. Cell. Proteomics* 3 692–703. 10.1074/mcp.M400003-MCP200 15073272

[B125] Lerman I. Davis B. A. Bertram T. M. Proudfoot J. Hauger R. L. Coe C. L. (2016). Posttraumatic stress disorder influences the nociceptive and intrathecal cytokine response to a painful stimulus in combat veterans. *Psychoneuroendocrinology* 73 99–108. 10.1016/j.psyneuen.2016.07.202 27490714

[B126] Létondor A. Buaud B. Vaysse C. Richard E. Layé S. Pallet V. (2016). EPA/DHA and vitamin a supplementation improves spatial memory and alleviates the age-related decrease in hippocampal rxrγ and kinase expression in rats. *Front. Aging Neurosci.* 8:103 10.3389/fnagi.2016.00103 PMC4860397 27242514

[B127] Levant B. Ozias M. K. Davis P. F. Winter M. Russell K. L. Carlson S. E. (2008). Decreased brain docosahexaenoic acid content produces neurobiological effects associated with depression: interactions with reproductive status in female rats. *Psychoneuroendocrinology* 33 1279–1292. 10.1016/j.psyneuen.2008.06.012 18707812 PMC2582014

[B128] Li Z. Ren W. Han X. Liu X. Wang G. Zhang M. (2015). ω3-polyunsaturated fatty acids suppress lipoprotein-associated phospholipase A2 expression in macrophages and animal models. *Mol. Nutr. Food Res.* 59 1771–1779. 10.1002/mnfr.201500022 26018800

[B129] Lin P.-Y. Huang S.-Y. Su K.-P. (2010). A meta-analytic review of polyunsaturated fatty acid compositions in patients with depression. *Biol. Psychiatry* 68 140–147. 10.1016/j.biopsych.2010.03.018 20452573

[B130] Liu J. J. Galfalvy H. C. Cooper T. B. Oquendo M. A. Grunebaum M. F. Mann J. J. (2013). Omega-3 polyunsaturated fatty acid (PUFA) status in major depressive disorder with comorbid anxiety disorders. *J. Clin. Psychiatry* 74 732–738. 10.4088/JCP.12m07970 23945451 PMC3905735

[B131] Lukiw W. J. Cui J.-G. Marcheselli V. L. Bodker M. Botkjaer A. Gotlinger K. (2005). A role for docosahexaenoic acid-derived neuroprotectin D1 in neural cell survival and Alzheimer disease. *J. Clin. Invest.* 115 2774–2783. 10.1172/JCI25420 16151530 PMC1199531

[B132] Ma D. Lu L. Boneva N. B. Warashina S. Kaplamadzhiev D. B. Mori Y. (2008). Expression of free fatty acid receptor GPR40 in the neurogenic niche of adult monkey hippocampus. *Hippocampus* 18 326–333. 10.1002/hipo.20393 18064707

[B133] Ma D. Tao B. Warashina S. Kotani S. Lu L. Kaplamadzhiev D. B. (2007). Expression of free fatty acid receptor GPR40 in the central nervous system of adult monkeys. *Neurosci. Res.* 58 394–401. 10.1016/j.neures.2007.05.001 17583366

[B134] Mackie K. (2008). Signaling via CNS cannabinoid receptors. *Mol. Cell. Endocrinol.* 286 S60–S65. 10.1016/j.mce.2008.01.022 18336996 PMC2435200

[B135] Madore C. Nadjar A. Delpech J.-C. Sere A. Aubert A. Portal C. (2014). Nutritional n-3 PUFAs deficiency during perinatal periods alters brain innate immune system and neuronal plasticity-associated genes. *Brain Behav. Immun.* 41 22–31. 10.1016/j.bbi.2014.03.021 24735929

[B136] Maes M. Smith R. Christophe A. Cosyns P. Desnyder R. Meltzer H. (1996). Fatty acid composition in major depression: decreased omega 3 fractions in cholesteryl esters and increased C20: 4 omega 6/C20:5 omega 3 ratio in cholesteryl esters and phospholipids. *J. Affect. Disord.* 38 35–46. 10.1016/0165-0327(95)00092-5 8735157

[B137] Makrides M. Gibson R. A. (2000). Long-chain polyunsaturated fatty acid requirements during pregnancy and lactation. *Am. J. Clin. Nutr.* 71 307S–311S. 10.1093/ajcn/71.1.307S 10617987

[B138] Manduca A. Bara A. Larrieu T. Lassalle O. Joffre C. Layé S. (2017). Amplification of mGlu 5 -endocannabinoid signaling rescues behavioral and synaptic deficits in a mouse model of adolescent and adult dietary polyunsaturated fatty acid imbalance. *J. Neurosci.* 37 6851–6868. 10.1523/JNEUROSCI.3516-16.2017 28630250 PMC6705718

[B139] Marangell L. B. Martinez J. M. Zboyan H. A. Kertz B. Kim H. F. S. Puryear L. J. (2003). A double-blind, placebo-controlled study of the omega-3 fatty acid docosahexaenoic acid in the treatment of major depression. *Am. J. Psychiatry* 160 996–998. 10.1176/appi.ajp.160.5.996 12727707

[B140] Marcheselli V. L. Hong S. Lukiw W. J. Tian X. H. Gronert K. Musto A. (2003). Novel docosanoids inhibit brain ischemia-reperfusion-mediated leukocyte infiltration and pro-inflammatory gene expression. *J. Biol. Chem.* 278 43807–43817. 10.1074/jbc.M305841200 12923200

[B141] Markhus M. W. Skotheim S. Graff I. E. Frøyland L. Braarud H. C. Stormark K. M. (2013). Low omega-3 index in pregnancy is a possible biological risk factor for postpartum depression. *PLoS One* 8:e67617. 10.1371/journal.pone.0067617 23844041 PMC3701051

[B142] Martins J. G. (2009). EPA but not DHA appears to be responsible for the efficacy of omega-3 long chain polyunsaturated fatty acid supplementation in depression: evidence from a meta-analysis of randomized controlled trials. *J. Am. Coll. Nutr.* 28 525–542. 10.1080/07315724.2009.10719785 20439549

[B143] Martins J. G. Bentsen H. Puri B. K. (2012). Eicosapentaenoic acid appears to be the key omega-3 fatty acid component associated with efficacy in major depressive disorder: a critique of Bloch and Hannestad and updated meta-analysis. *Mol. Psychiatry* 17 1144–1149. 10.1038/mp.2012.25 22488258

[B144] Mathias R. A. Pani V. Chilton F. H. (2014). Genetic variants in the fads gene: implications for dietary recommendations for Fatty Acid intake. *Curr. Nutr. Rep.* 3 139–148. 10.1007/s13668-014-0079-1 24977108 PMC4070521

[B145] Matsuoka Y. Nishi D. Hamazaki K. Yonemoto N. Matsumura K. Noguchi H. (2015). Docosahexaenoic acid for selective prevention of posttraumatic stress disorder among severely injured patients: a randomized, placebo-controlled trial. *J. Clin. Psychiatry* 76 e1015–e1022. 10.4088/JCP.14m09260 26335087

[B146] Matsuoka Y. J. Hamazaki K. Nishi D. Hamazaki T. (2016). Change in blood levels of eicosapentaenoic acid and posttraumatic stress symptom: a secondary analysis of data from a placebo-controlled trial of omega3 supplements. *J. Affect. Disord.* 205 289–291. 10.1016/j.jad.2016.08.005 27552592

[B147] McLaughlin R. J. Hill M. N. Gorzalka B. B. (2014). A critical role for prefrontocortical endocannabinoid signaling in the regulation of stress and emotional behavior. *Neurosci. Biobehav. Rev.* 42 116–131. 10.1016/j.neubiorev.2014.02.006 24582908

[B148] McNamara R. K. Carlson S. E. (2006). Role of omega-3 fatty acids in brain development and function: potential implications for the pathogenesis and prevention of psychopathology. *Prostaglandins Leukot. Essent. Fatty Acids* 75 329–349. 10.1016/j.plefa.2006.07.010 16949263

[B149] McNamara R. K. Hahn C.-G. Jandacek R. Rider T. Tso P. Stanford K. E. (2007). Selective deficits in the omega-3 fatty acid docosahexaenoic acid in the postmortem orbitofrontal cortex of patients with major depressive disorder. *Biol. Psychiatry* 62 17–24. 10.1016/j.biopsych.2006.08.026 17188654

[B150] McNamara R. K. Jandacek R. Rider T. Tso P. Stanford K. E. Hahn C.-G. (2008a). Deficits in docosahexaenoic acid and associated elevations in the metabolism of arachidonic acid and saturated fatty acids in the postmortem orbitofrontal cortex of patients with bipolar disorder. *Psychiatry Res.* 160 285–299. 10.1016/j.psychres.2007.08.021 18715653 PMC2620106

[B151] McNamara R. K. Liu Y. Jandacek R. Rider T. Tso P. (2008b). The aging human orbitofrontal cortex: decreasing polyunsaturated fatty acid composition and associated increases in lipogenic gene expression and stearoyl-CoA desaturase activity. *Prostaglandins Leukot. Essent. Fatty Acids* 78 293–304. 10.1016/j.plefa.2008.04.001 18499418 PMC2494852

[B152] McNamara R. K. Jandacek R. Tso P. Dwivedi Y. Ren X. Pandey G. N. (2013). Lower docosahexaenoic acid concentrations in the postmortem prefrontal cortex of adult depressed suicide victims compared with controls without cardiovascular disease. *J. Psychiatr. Res.* 47 1187–1191. 10.1016/j.jpsychires.2013.05.007 23759469 PMC3710518

[B153] McNamara R. K. Liu Y. (2011). Reduced expression of fatty acid biosynthesis genes in the prefrontal cortex of patients with major depressive disorder. *J. Affect. Disord.* 129 359–363. 10.1016/j.jad.2010.08.021 20863572 PMC3023006

[B154] McNamara R. K. Strimpfel J. Jandacek R. Rider T. Tso P. Welge J. A. (2014). Detection and treatment of long-chain omega-3 fatty acid deficiency in adolescents with SSRI-Resistant Major Depressive Disorder. *Pharmanutrition* 2 38–46. 10.1016/j.phanu.2014.02.002 24772386 PMC3998067

[B155] Ménard C. Pfau M. L. Hodes G. E. Russo S. J. (2017). Immune and neuroendocrine mechanisms of stress vulnerability and resilience. *Neuropsychopharmacology* 42 62–80. 10.1038/npp.2016.90 27291462 PMC5143517

[B156] Meneses J. A. de Trugilho L. A. Lima S. A. Freitas A. C. F. Melo H. S. Ferreira M. R. (2017). The influence of a diet based on flaxseed, an omega-3 source, during different developmental periods, on the blood pressure of rats submitted to stress. *J. Matern. Fetal. Neonatal Med.* 10.1080/14767058.2017.1407309 [Epub ahead of print]. 29187002

[B157] Messamore E. McNamara R. K. (2016). Detection and treatment of omega-3 fatty acid deficiency in psychiatric practice: rationale and implementation. *Lipids Health Dis.* 15:25. 10.1186/s12944-016-0196-5 26860589 PMC4748485

[B158] Mingam R. Moranis A. Bluthé R.-M. De Smedt-Peyrusse V. Kelley K. W. Guesnet P. (2008). Uncoupling of interleukin-6 from its signalling pathway by dietary n-3-polyunsaturated fatty acid deprivation alters sickness behaviour in mice. *Eur. J. Neurosci.* 28 1877–1886. 10.1111/j.1460-9568.2008.06470.x 18973601 PMC2769572

[B159] Mischoulon D. Nierenberg A. A. Schettler P. J. Kinkead B. L. Fehling K. Martinson M. A. (2015). A double-blind, randomized controlled clinical trial comparing eicosapentaenoic acid versus docosahexaenoic acid for depression. *J. Clin. Psychiatry* 76 54–61. 10.4088/JCP.14m08986 25272149 PMC11708509

[B160] Mocking R. J. T. Harmsen I. Assies J. Koeter M. W. J. Ruhé H. G. Schene A. H. (2016). Meta-analysis and meta-regression of omega-3 polyunsaturated fatty acid supplementation for major depressive disorder. *Transl. Psychiatry* 6:e756. 10.1038/tp.2016.29 26978738 PMC4872453

[B161] Mocking R. J. T. Ruhé H. G. Assies J. Lok A. Koeter M. W. J. Visser I. (2013). Relationship between the hypothalamic-pituitary-adrenal-axis and fatty acid metabolism in recurrent depression. *Psychoneuroendocrinology* 38 1607–1617. 10.1016/j.psyneuen.2013.01.013 23465556

[B162] Morena M. Patel S. Bains J. S. Hill M. N. (2015). Neurobiological interactions between stress and the endocannabinoid system. *Neuropsychopharmacology* 41 80–102. 10.1038/npp.2015.166 26068727 PMC4677118

[B163] Morgese M. G. Tucci P. Mhillaj E. Bove M. (2016). Lifelong nutritional Omega-3 deficiency evokes depressive-like state through soluble beta amyloid. *Mol. Neurobiol.* 54 2079–2089. 10.1007/s12035-016-9809-2 26924315 PMC5355522

[B164] Müller C. P. Reichel M. Mühle C. Rhein C. Gulbins E. Kornhuber J. (2015). Brain membrane lipids in major depression and anxiety disorders. *Biochim. Biophys. Acta Mol. Cell Biol. Lipids* 1851 1052–1065. 10.1016/j.bbalip.2014.12.014 25542508

[B165] Nakamoto K. Aizawa F. Nishinaka T. Tokuyama S. (2015). Regulation of prohormone convertase 2 protein expression via GPR40/FFA1 in the hypothalamus. *Eur. J. Pharmacol.* 762 459–463. 10.1016/j.ejphar.2015.06.013 26071852

[B166] Naliwaiko K. Araújo R. L. E. da Fonseca R. V. Castilho J. C. Andreatini R. Bellissimo I. (2004). Effects of fish oil on the central nervous system: a new potential antidepressant? *Nutr. Neurosci.* 7 91–99. 10.1080/10284150410001704525 15279495

[B167] Nascimento L. F. R. Souza G. F. P. Morari J. Barbosa G. O. Solon C. Moura R. F. (2016). n-3 Fatty Acids induce neurogenesis of predominantly pomc-expressing cells in the hypothalamus. *Diabetes Metab. Res. Rev.* 65 673–686. 10.2337/db15-0008 26512023

[B168] Nemets B. Stahl Z. Belmaker R. H. (2002). Addition of omega-3 fatty acid to maintenance medication treatment for recurrent unipolar depressive disorder. *Am. J. Psychiatry* 159 477–479. 10.1176/appi.ajp.159.3.477 11870016

[B169] Nemets H. Nemets B. Apter A. Bracha Z. Belmaker R. H. (2006). Omega-3 treatment of childhood depression: a controlled, double-blind pilot study. *Am. J. Psychiatry* 163 1098–1100. 10.1176/ajp.2006.163.6.1098 16741212

[B170] Newton T. L. Fernandez-Botran R. Miller J. J. Burns V. E. (2014). Interleukin-6 and soluble interleukin-6 receptor levels in posttraumatic stress disorder: associations with lifetime diagnostic status and psychological context. *Biol. Psychol.* 99 150–159. 10.1016/j.biopsycho.2014.03.009 24695006 PMC4059765

[B171] Nieminen L. R. G. Makino K. K. Mehta N. Virkkunen M. Kim H. Y. Hibbeln J. R. (2006). Relationship between omega-3 fatty acids and plasma neuroactive steroids in alcoholism, depression and controls. *Prostaglandins Leukot. Essent. Fatty Acids* 75 309–314. 10.1016/j.plefa.2006.07.012 16959481

[B172] Nishinaka T. Yamashita T. Nakamoto K. Kasuya F. Tokuyama S. (2014). Involvement of the long-chain fatty acid receptor GPR40 in depression-related behavior. *J. Pharmacol. Sci.* 125 112–115. 10.1254/jphs.14001SC 24758921

[B173] Orr S. K. Palumbo S. Bosetti F. Mount H. T. Kang J. X. Greenwood C. E. (2013). Unesterified docosahexaenoic acid is protective in neuroinflammation. *J. Neurochem.* 127 378–393. 10.1111/jnc.12392 23919613 PMC4068707

[B174] Otoki Y. Hennebelle M. Levitt A. J. Nakagawa K. Swardfager W. Taha A. Y. (2017). Plasma phosphatidylethanolamine and triacylglycerol fatty acid concentrations are altered in major depressive disorder patients with seasonal pattern. *Lipids* 52 559–571. 10.1007/s11745-017-4254-1 28439746

[B175] Pan J. P. Zhang H. Q. Wei-Wang Guo Y. F. Na-Xiao Cao X. H. (2011). Some subtypes of endocannabinoid/endovanilloid receptors mediate docosahexaenoic acid-induced enhanced spatial memory in rats. *Brain Res.* 1412 18–27. 10.1016/j.brainres.2011.07.015 21803345

[B176] Park H. G. Park W. J. Kothapalli K. S. D. Brenna J. T. (2015). The fatty acid desaturase 2 (FADS2) gene product catalyzes Δ4 desaturation to yield n-3 docosahexaenoic acid and n-6 docosapentaenoic acid in human cells. *FASEB J.* 29 3911–3919. 10.1096/fj.15-271783 26065859 PMC4550368

[B177] Parker G. Hegarty B. Granville-Smith I. Ho J. Paterson A. Gokiert A. (2015). Is essential fatty acid status in late pregnancy predictive of post-natal depression? *Acta Psychiatr. Scand.* 131 148–156. 10.1111/acps.12321 25131141

[B178] Parletta N. Zarnowiecki D. Cho J. Wilson A. Procter N. Gordon A. (2016). People with schizophrenia and depression have a low omega-3 index. *Prostaglandins Leukot. Essent. Fatty Acids* 110 42–47. 10.1016/j.plefa.2016.05.007 27255642

[B179] Passos I. C. Vasconcelos-Moreno M. P. Costa L. G. Kunz M. Brietzke E. Quevedo J. (2015). Inflammatory markers in post-traumatic stress disorder: a systematic review, meta-analysis, and meta-regression. *Lancet Psychiatry* 2 1002–1012. 10.1016/S2215-0366(15)00309-0 26544749

[B180] Peet M. Horrobin D. F. (2002). A dose-ranging study of the effects of ethyl-eicosapentaenoate in patients with ongoing depression despite apparently adequate treatment with standard drugs. *Arch. Gen. Psychiatry* 59 913–919. 10.1001/archpsyc.59.10.913 12365878

[B181] Pettit L. K. Varsanyi C. Tadros J. Vassiliou E. (2013). Modulating the inflammatory properties of activated microglia with docosahexaenoic acid and aspirin. *Lipids Health Dis.* 12:16. 10.1186/1476-511X-12-16 23398903 PMC3663775

[B182] Piomelli D. Sasso O. (2014). Peripheral gating of pain signals by endogenous lipid mediators. *Nat. Neurosci.* 17 164–174. 10.1038/nn.3612 24473264 PMC4020413

[B183] Raeder M. B. Steen V. M. Vollset S. E. Bjelland I. (2007). Associations between cod liver oil use and symptoms of depression: the hordaland health study. *J. Affect. Disord.* 101 245–249. 10.1016/j.jad.2006.11.006 17184843

[B184] Raison C. L. Miller A. H. (2011). Is depression an inflammatory disorder? *Curr. Psychiatry Rep.* 13 467–475. 10.1007/s11920-011-0232-0 21927805 PMC3285451

[B185] Rapaport M. H. Nierenberg A. A. Schettler P. J. Kinkead B. Cardoos A. Walker R. (2016). Inflammation as a predictive biomarker for response to omega-3 fatty acids in major depressive disorder: a proof-of-concept study. *Mol. Psychiatry* 21 71–79. 10.1038/mp.2015.22 25802980 PMC4581883

[B186] Rees A.-M. Austin M.-P. Parker G. B. (2008). Omega-3 fatty acids as a treatment for perinatal depression: randomized double-blind placebo-controlled trial. *Aust. N. Z. J. Psychiatry* 42 199–205. 10.1080/00048670701827267 18247194

[B187] Rey C. Nadjar A. Buaud B. Vaysse C. Aubert A. Pallet V. (2016). Resolvin D1 and E1 promote resolution of inflammation in microglial cells in vitro. *Brain Behav. Immun.* 55 249–259. 10.1016/j.bbi.2015.12.013 26718448

[B188] Rosenberger T. A. Villacreses N. E. Hovda J. T. Bosetti F. Weerasinghe G. Wine R. N. (2004). Rat brain arachidonic acid metabolism is increased by a 6-day intracerebral ventricular infusion of bacterial lipopolysaccharide. *J. Neurochem.* 88 1168–1178. 10.1046/j.1471-4159.2003.02246.x 15009672

[B189] Rush A. J. Trivedi M. H. Wisniewski S. R. Nierenberg A. A. Stewart J. W. Warden D. (2006a). Acute and longer-term outcomes in depressed outpatients requiring one or several treatment steps: a STAR^∗^D report. *Am. J. Psychiatry* 163 1905–1917. 10.1176/ajp.2006.163.11.1905 17074942

[B190] Rush A. J. Trivedi M. H. Wisniewski S. R. Stewart J. W. Nierenberg A. A. Thase M. E. (2006b). Bupropion-SR, sertraline, or venlafaxine-XR after failure of SSRIs for depression. *N. Engl. J. Med.* 354 1231–1242. 10.1056/NEJMoa052963 16554525

[B191] Saito M. Klibert J. Langhinrichsen-Rohling J. (2013). Suicide proneness in American and Japanese college students: associations with suicide acceptability and emotional expressivity. *Death Stud.* 37 848–865. 10.1080/07481187.2012.699910 24517594

[B192] Sakamoto T. Cansev M. Wurtman R. J. (2007). Oral supplementation with docosahexaenoic acid and uridine-5’-monophosphate increases dendritic spine density in adult gerbil hippocampus. *Brain Res.* 1182 50–59. 10.1016/j.brainres.2007.08.089 17950710 PMC2140951

[B193] Sandi C. Richter-Levin G. (2009). From high anxiety trait to depression: a neurocognitive hypothesis. *Trends Neurosci.* 32 312–320. 10.1016/j.tins.2009.02.004 19409624

[B194] Sastry P. S. (1985). Lipids of nervous tissue: composition and metabolism. *Prog. Lipid Res.* 24 69–176. 10.1016/0163-7827(85)90011-6 3916238

[B195] Saunders E. F. H. Ramsden C. E. Sherazy M. S. Gelenberg A. J. Davis J. M. Rapoport S. I. (2016). Reconsidering dietary polyunsaturated fatty acids in bipolar disorder: a translational picture. *J. Clin. Psychiatry* 77 e1342–e1347. 10.4088/JCP.15com10431 27788314 PMC6093189

[B196] Schizophrenia Psychiatric Genome-Wide Association Study (GWAS) Consortium (2011). Genome-wide association study identifies five new schizophrenia loci. *Nat. Genet.* 43 969–976. 10.1038/ng.940 21926974 PMC3303194

[B197] Scola G. Versace A. Metherel A. H. Monsalve-Castro L. A. Phillips M. L. Bazinet R. P. (2018). Alterations in peripheral fatty acid composition in bipolar and unipolar depression. *J. Affect. Disord.* 233 86–91. 10.1016/j.jad.2017.12.025 29336895

[B198] Serhan C. N. (2014). Pro-resolving lipid mediators are leads for resolution physiology. *Nature* 510 92–101. 10.1038/nature13479 24899309 PMC4263681

[B199] Serhan C. N. (2017). Discovery of specialized pro-resolving mediators marks the dawn of resolution physiology and pharmacology. *Mol. Aspects Med.* 58 1–11. 10.1016/j.mam.2017.03.001 28263773 PMC5582020

[B200] Serhan C. N. Fredman G. Yang R. Karamnov S. Belayev L. S. Bazan N. G. (2011). Novel proresolving aspirin-triggered DHA pathway. *Chem. Biol.* 18 976–987. 10.1016/j.chembiol.2011.06.008 21867913 PMC3164791

[B201] Shelton R. C. Osuntokun O. Heinloth A. N. Corya S. A. (2010). Therapeutic options for treatment-resistant depression. *CNS Drugs* 24 131–161. 10.2165/11530280-000000000-00000 20088620

[B202] Shi Z. Ren H. Huang Z. Peng Y. He B. Yao X. (2017). Fish oil prevents lipopolysaccharide-induced depressive-like behavior by inhibiting neuroinflammation. *Mol. Neurobiol.* 54 7327–7334. 10.1007/s12035-016-0212-9 27815837

[B203] Silvers K. M. Scott K. M. (2002). Fish consumption and self-reported physical and mental health status. *Public Health Nutr.* 5 427–431. 10.1079/PHNPHN2001308 12003654

[B204] Simopoulos A. P. (1991). Omega-3 fatty acids in health and disease and in growth and development. *Am. J. Clin. Nutr.* 54 438–463. 10.1093/ajcn/54.3.438 1908631

[B205] Song C. Li X. Leonard B. E. Horrobin D. F. (2003). Effects of dietary n-3 or n-6 fatty acids on interleukin-1beta-induced anxiety, stress, and inflammatory responses in rats. *J. Lipid Res.* 44 1984–1991. 10.1194/jlr.M300217-JLR200 12837849

[B206] Spector A. A. Kim H.-Y. (2015). Discovery of essential fatty acids. *J. Lipid Res.* 56 11–21. 10.1194/jlr.R055095 25339684 PMC4274059

[B207] Su K.-P. Huang S.-Y. Chiu C.-C. Shen W. W. (2003). Omega-3 fatty acids in major depressive disorder. A preliminary double-blind, placebo-controlled trial. *Eur. Neuropsychopharmacol.* 13 267–271. 10.1016/S0924-977X(03)00032-4 12888186

[B208] Sublette M. E. Ellis S. P. Geant A. L. Mann J. J. (2011). Meta-analysis of the effects of eicosapentaenoic acid (EPA) in clinical trials in depression. *J. Clin. Psychiatry* 72 1577–1584. 10.4088/JCP.10m06634 21939614 PMC3534764

[B209] Sublette M. E. Vaquero C. Baca-Garcia E. Pachano G. Huang Y.-Y. Oquendo M. A. (2016). Lack of association of SNPs from the FADS1-FADS2 gene cluster with major depression or suicidal behavior. *Psychiatr. Genet.* 26 81–86. 10.1097/YPG.0000000000000111 26513616 PMC4764474

[B210] Sugiura T. Kondo S. Sukagawa A. Nakane S. Shinoda A. Itoh K. (1995). 2-Arachidonoylglycerol: a possible endogenous cannabinoid receptor ligand in brain. *Biochem. Biophys. Res. Commun.* 215 89–97. 10.1006/bbrc.1995.2437 7575630

[B211] Taha A. Y. Blanchard H. C. Cheon Y. Ramadan E. Chen M. Chang L. (2017). Dietary linoleic acid lowering reduces lipopolysaccharide-induced increase in brain arachidonic acid metabolism. *Mol. Neurobiol.* 54 4303–4315. 10.1007/s12035-016-9968-1 27339880 PMC5843819

[B212] Taha A. Y. Hennebelle M. Yang J. Zamora D. Rapoport S. I. Hammock B. D. (2016). Regulation of rat plasma and cerebral cortex oxylipin concentrations with increasing levels of dietary linoleic acid. *Prostaglandins Leukot. Essent. Fatty Acids* 10.1016/j.plefa.2016.05.004 [Epub ahead of print]. 27282298 PMC5106341

[B213] Takeuchi T. Iwanaga M. Harada E. (2003). Possible regulatory mechanism of DHA-induced anti-stress reaction in rats. *Brain Res.* 964 136–143. 10.1016/S0006-8993(02)04113-6 12573522

[B214] Tanskanen A. Hibbeln J. R. Tuomilehto J. Uutela A. Haukkala A. Viinamäki H. (2001). Fish consumption and depressive symptoms in the general population in Finland. *Psychiatr. Serv.* 52 529–531. 10.1176/appi.ps.52.4.529 11274502

[B215] Tejera N. Vauzour D. Betancor M. B. Sayanova O. Usher S. Cochard M. (2016). A transgenic *Camelina sativa* seed oil effectively replaces fish oil as a dietary source of eicosapentaenoic acid in mice. *J. Nutr.* 146 227–235. 10.3945/jn.115.223941 26791554 PMC4725436

[B216] Thomazeau A. Bosch-Bouju C. Manzoni O. Layé S. (2017). Nutritional n-3 PUFA deficiency abolishes endocannabinoid gating of hippocampal long-term potentiation. *Cereb. Cortex* 27 2571–2579. 10.1093/cercor/bhw052 26946127

[B217] Tiemeier H. van Tuijl H. R. Hofman A. Kiliaan A. J. Breteler M. M. B. (2003). Plasma fatty acid composition and depression are associated in the elderly: the rotterdam study. *Am. J. Clin. Nutr.* 78 40–46. 10.1093/ajcn/78.1.40 12816769

[B218] Timonen M. Horrobin D. Jokelainen J. Laitinen J. Herva A. Räsänen P. (2004). Fish consumption and depression: the Northern Finland 1966 birth cohort study. *J. Affect. Disord.* 82 447–452. 10.1016/j.jad.2004.02.002 15555697

[B219] Tremoleda J. L. Thau-Zuchman O. Davies M. Foster J. Khan I. Vadivelu K. C. (2016). In vivo PET imaging of the neuroinflammatory response in rat spinal cord injury using the TSPO tracer [^18^F]GE-180 and effect of docosahexaenoic acid. *Eur. J. Nucl. Med. Mol. Imaging* 43 1710–1722. 10.1007/s00259-016-3391-8 27154521 PMC4932147

[B220] Uyanik V. Tuglu C. Gorgulu Y. Kunduracilar H. Uyanik M. S. (2015). Assessment of cytokine levels and hs-CRP in bipolar I disorder before and after treatment. *Psychiatry Res.* 228 386–392. 10.1016/j.psychres.2015.05.078 26160203

[B221] Vaz J. D. S. Farias D. R. Adegboye A. R. A. Nardi A. E. Kac G. (2017). Omega-3 supplementation from pregnancy to postpartum to prevent depressive symptoms: a randomized placebo-controlled trial. *BMC Pregnancy Childbirth* 17:180. 10.1186/s12884-017-1365-x 28599630 PMC5466796

[B222] Watanabe S. Doshi M. Hamazaki T. (2003). n-3 Polyunsaturated fatty acid (PUFA) deficiency elevates and n-3 PUFA enrichment reduces brain 2-arachidonoylglycerol level in mice. *Prostaglandins Leukot. Essent. Fatty Acids* 69 51–59. 10.1016/S0952-3278(03)00056-5 12878451

[B223] WHO (2015) *Suicide.* Available at: http://www.who.int/mediacentre/factsheets/fs398/en/

[B224] Wietrzych-Schindler M. Szyszka-Niagolov M. Ohta K. Endo Y. Pérez E. de Lera A. R. (2011). Retinoid x receptor gamma is implicated in docosahexaenoic acid modulation of despair behaviors and working memory in mice. *Biol. Psychiatry* 69 788–794. 10.1016/j.biopsych.2010.12.017 21334601

[B225] Wilson R. I. Nicoll R. A. (2002). Endocannabinoid signaling in the brain. *Science* 296 678–682. 10.1126/science.1063545 11976437

[B226] Wilson R. I. Nicoll R. A. (2001). Endogenous cannabinoids mediate retrograde signalling at hippocampal synapses. *Nature* 410 588–592. 10.1038/35069076 11279497

[B227] Wood J. T. Williams J. S. Pandarinathan L. Janero D. R. Lammi-Keefe C. J. Makriyannis A. (2010). Dietary docosahexaenoic acid supplementation alters select physiological endocannabinoid-system metabolites in brain and plasma. *J. Lipid Res.* 51 1416–1423. 10.1194/jlr.M002436 20071693 PMC3035504

[B228] Yamashima T. (2008). A putative link of PUFA, GPR40 and adult-born hippocampal neurons for memory. *Prog. Neurobiol.* 84 105–115. 10.1016/j.pneurobio.2007.11.002 18191887

[B229] Yamauchi T. Fujita T. Tachimori H. Takeshima T. Inagaki M. Sudo A. (2013). Age-adjusted relative suicide risk by marital and employment status over the past 25 years in Japan. *J. Public Health* 35 49–56. 10.1093/pubmed/fds054 22789751

[B230] Yehuda S. Rabinovitz S. Mostofsky D. I. (2005). Mixture of essential fatty acids lowers test anxiety. *Nutr. Neurosci.* 8 265–267. 10.1080/10284150500445795 16491653

[B231] Zainuddin M. S. A. Thuret S. (2012). Nutrition, adult hippocampal neurogenesis and mental health. *Br. Med. Bull.* 103 89–114. 10.1093/bmb/lds021 22833570

[B232] Zimmer R. Riemer T. Rauch B. Schneider S. Schiele R. Gohlke H. (2013). Effects of 1-Year treatment with highly purified omega-3 fatty acids on depression after myocardial infarction. *J. Clin. Psychiatry* 74 e1037–e1045. 10.4088/JCP.13m08453 24330904

